# Post-Stroke Longitudinal Alterations of Inter-Hemispheric Correlation and Hemispheric Dominance in Mouse Pre-Motor Cortex

**DOI:** 10.1371/journal.pone.0146858

**Published:** 2016-01-11

**Authors:** Fabio Vallone, Stefano Lai, Cristina Spalletti, Alessandro Panarese, Claudia Alia, Silvestro Micera, Matteo Caleo, Angelo Di Garbo

**Affiliations:** 1 Institute of Biophysics, CNR, Pisa, Italy; 2 Translational Neural Engineering Area, The Biorobotics Institute, Scuola Superiore Sant’Anna, Pisa, Italy; 3 Neuroscience Institute, CNR, Pisa, Italy; 4 Life Science Institute, Scuola Superiore Sant’Anna, Pisa, Italy; 5 Scuola Normale Superiore, Pisa, Italy; 6 Bertarelli Foundation Chair in Translational Neuroengineering Center for Neuroprosthetics and Institute of Bioengineering School of Engineering Ecole Polytechnique Federale de Lausanne (EPFL), Lausanne, Switzerland; University of Münster, GERMANY

## Abstract

**Purpose:**

Limited restoration of function is known to occur spontaneously after an ischemic injury to the primary motor cortex. Evidence suggests that Pre-Motor Areas (PMAs) may “take over” control of the disrupted functions. However, little is known about functional reorganizations in PMAs. Forelimb movements in mice can be driven by two cortical regions, Caudal and Rostral Forelimb Areas (CFA and RFA), generally accepted as primary motor and pre-motor cortex, respectively. Here, we examined longitudinal changes in functional coupling between the two RFAs following unilateral photothrombotic stroke in CFA (mm from Bregma: +0.5 anterior, +1.25 lateral).

**Methods:**

Local field potentials (LFPs) were recorded from the RFAs of both hemispheres in freely moving injured and naïve mice. Neural signals were acquired at 9, 16 and 23 days after surgery (sub-acute period in stroke animals) through one bipolar electrode per hemisphere placed in the center of RFA, with a ground screw over the occipital bone. LFPs were pre-processed through an efficient method of artifact removal and analysed through: spectral,cross-correlation, mutual information and Granger causality analysis.

**Results:**

Spectral analysis demonstrated an early decrease (day 9) in the alpha band power in both the RFAs. In the late sub-acute period (days 16 and 23), inter-hemispheric functional coupling was reduced in ischemic animals, as shown by a decrease in the cross-correlation and mutual information measures. Within the gamma and delta bands, correlation measures were already reduced at day 9. Granger analysis, used as a measure of the symmetry of the inter-hemispheric causal connectivity, showed a less balanced activity in the two RFAs after stroke, with more frequent oscillations of hemispheric dominance.

**Conclusions:**

These results indicate robust electrophysiological changes in PMAs after stroke. Specifically, we found alterations in transcallosal connectivity, with reduced inter-hemispheric functional coupling and a fluctuating dominance pattern. These reorganizations may underlie vicariation of lost functions following stroke.

## Introduction

Stroke is one of the main causes of death and disability worldwide [1]. Post-stroke deficits depend on both the location and size of the infarcted brain region. Ischemic infarctions in motor areas are particularly disabling, significantly impacting the execution of many activities of daily life (ADLs). Significant but limited spontaneous restoration of motor function occurs after injury and several studies have aimed at understanding the mechanisms underlying this complex process. In this context, many studies have investigated the interactions between brain hemispheres before and after stroke. Indeed, neural activity in brain motor areas is functionally coupled between the two hemispheres [2] and the lateralization of neural activity during movements is likely to be related to inter-hemispheric inhibition between motor areas exerted via transcallosal connections [3]. During recovery from a unilateral brain injury, patients in several clinical studies have shown changes in these inter-hemispheric influences [4, 5] which are thought to be caused by an imbalance of the mutual inter-hemispheric inhibition between the homotopic motor cortices [6]. It has been proposed that this inter-hemispheric disequilibrium could be an obstacle for motor recovery [6–8]. Following lesions to the Primary Motor Cortex (M1), regions adjacent to the infarct, such as Pre-Motor Areas (PMAs), also reorganize extensively [9]. The role of these brain map changes in recovery/compensation (and possibly, maladaptive plasticity) following injury is still incompletely understood [10].

In studies on monkeys, Liu and Rouiller found a significant regain of grasping ability after a lesion in the hand representation [11]. Acute pharmacological inactivation of the ipsi-lesional PMA abrogated completely this functional recovery, thus indicating a possible key role of PMAs in functional recovery after lesion in M1 [11].

In humans and monkeys, a detailed subdivision of M1 and PMAs is reported, based on cytoarchitecture, connectivity and functional properties [12, 13]. Similarly, a subdivision of rodent forelimb motor cortex has been proposed in rats [14] and, more recently, in mouse [15], based on the underlying cytoarchitecture and electrophysiological characteristics thus identifying two forelimb motor areas: the Caudal and the Rostral forelimb area (CFA and RFA). Many studies support the idea that RFA is putatively homologous to the primate premotor cortex, based on afferent and efferent projections and on the features of spiking activity during skilled forelimb movements [16–19]. Indeed, during skilled reaching, the discharge of RFA neurons is more dependent (as compared to CFA neurons) to changes in the behavioral context such as reward delivery [19]. Moreover the RFA, but not CFA, exhibits dense reciprocal connections with the insular cortex. RFA also receives more thalamic input from the ventromedial nucleus than from the ventrolateral nucleus, similar to monkey premotor areas [16]. Conversely, the CFA is analogous to the “old” M1 described in monkeys [20], whose descending commands must use the integrative mechanisms of the spinal cord to generate motoneuron activity. Altogether, the available evidences indicate that the rodent CFA corresponds to a primary motor area, while the more anterior RFA is thought to be more similar to a PMA [15, 17, 21, 22].

In the present study, we used a photothrombotic model of stroke in CFA that allows to generate reproducible, focal cortical infarcts, thus facilitating the evaluation of the plastic reorganization in the spared circuits adjacent to the lesion, such as RFA. This model has limitations, considering that variability in lesion size and location is a major confound in the clinical treatment of stroke. On the other hand, human strokes are mostly small in size [23]. In this context, the photothrombotic model offers standardized lesions that are suited for quantitative analyses and investigations about inter-hemispheric connectivity and plastic phenomena after brain injury.

In both humans and animals models [24], inter-hemispheric interactions have been investigated by means of functional magnetic resonance imaging (fMRI). The fMRI allows for quantification of the connectivity level between the neuronal activities of different brain regions by measuring the coupling among BOLD signals [25, 26]. A second suitable technique for such investigations is the multichannel EEG recording which is minimally invasive and allows for a higher temporal resolution measurement of the functional connectivity of different cortical regions [27]. However, animal models offer the further possibility to intra-cortically record the local neural activity, i.e. Local Field Potentials (LFPs), generated by transmembrane current flow in ensembles of neurons close to the site of electrode insertion [28, 29]. These recordings can also be performed in freely-moving behaviour, which is a spontaneous activity involving forelimbs where mice can move with the lowest constriction granted by the experimental conditions, thus reflecting a self-determined behavior [30]. This setup is largely used in different fields of research [31–33], in particular in studies about stroke [31].

In this work, we recorded LFPs from both RFAs in freely moving mice at different time intervals after a cortical lesion in CFA and we used several quantitative methods of time series analysis (spectral, cross-correlation, mutual information and Granger causality analysis) to monitor longitudinal changes in neural activity. Our purpose was to characterize the electrophysiological changes within and among Pre-Motor Areas, that potentially participate in recovery of forelimb function after an ischemic injury in CFA. [16, 34].

## Methods

### Ethics statement

All procedures were performed according to the guidelines of the Italian Ministry of Health for care and maintenance of laboratory animals, and in strict compliance with the European Community Directive n. 2010/63/EU on the protection of animals used for scientific purposes. Animal experimentation at the CNR Neuroscience Institute was approved by the Italian Ministry of Health (authorization #129/20002A). Specifically, the experiments described in this study were authorized by the Italian Ministry of Health via decree # 45/2014-B, released on Feb 10, 2014. All surgical procedures were performed under deep anesthesia and all efforts were made to ameliorate suffering of animals. At the end of the recordings, animals were killed by cervical dislocation, and brains were dissected for histological controls.

### Animals

A total number of 9 and 12 C57BL6-J mice were used for Intra-cortical microstimulation (ICMS) and LFP recordings, respectively. Age is one of the principal risk factors for stroke in humans [35], therefore we decided to use fully adult animals (6 months) for this study, in order to mimic human post-stroke conditions. For the optogenetic stimulation experiments, Thy1-ChR2 Transgenic mice (B6.Cg-Tg (Thy1-ChR2/EYFP)18Cfng/J, Jackson Laboratories, USA) were used.

### Intra-cortical microstimulation and optogenetic experiments

A stimulation protocol similar to previously reported experiments was used [15, 36]. Briefly, mice were anesthetized with an initial cocktail of ketamine (100 mg/kg, i.p.) and xylazine (10 mg/kg, i.p.). A tungsten microelectrode (1 M*Ω*, FHC, USA) was inserted slowly in the brain at 700 *μ*m of depth for each stimulation point following a grid with nodes spaced 250 *μ*m and including both the CFA and the RFA.

**Fig 1:**
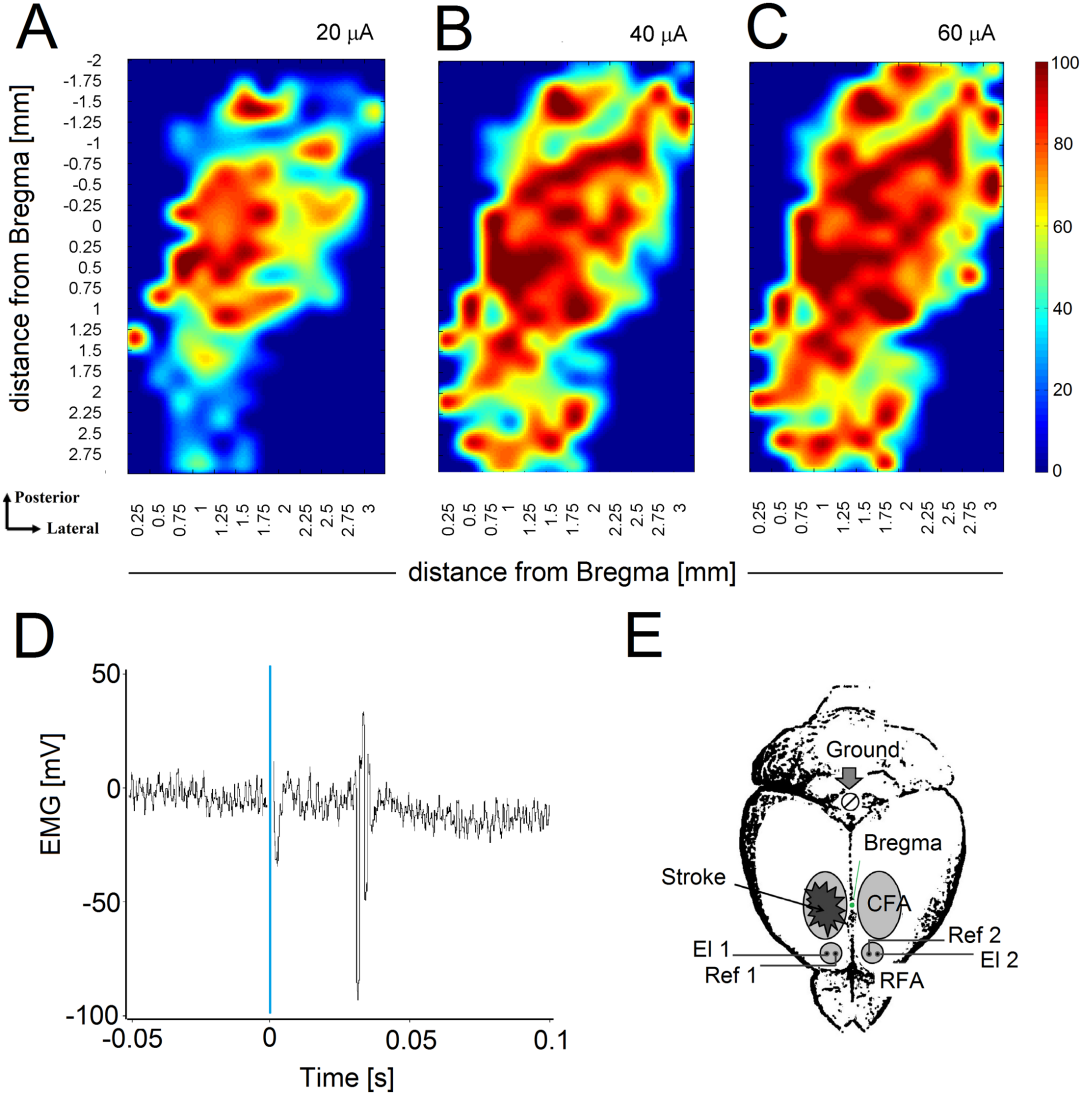
ICMS map, optogenetic MEP recording and schematic of stroke location and electrodes position. A-C) Mean maps of forelimb movements evoked in all animals at 20,40 and 60 *μ*A current threshold in naïve mice (*n* = 9). Coordinates for lesion and electrode implantation were based on this map. The color bar represents the probability to elicit a forelimb movement after stimulation of a specific site. D) Representative MEP recorded from the forelimb contra-lateral to the stimulated RFA. The blue line at time 0 represents the light stimulation. E) Stroke was unilaterally induced in CFA whereas bipolar electrodes were inserted in both RFAs: recording (El1 and El2) and reference (Ref1 and Ref2) electrodes. A surgical screw (Ground) was placed in the occipital bone and used as ground reference.

At each penetration, a 40 ms train of 13 200 *μ*s monophasic cathodal pulses was delivered at 350 Hz from an electrically isolated, constant current stimulator (World Precision Instruments Inc., USA) driven by a electronic board (National Instruments Corp, USA) through a custom-made interface implemented in Lab-View (National Instruments Corp, USA) at a rate 1 Hz. The amplitude of the pulses was increased from a minimum of 20 to a maximum of 60 *μ*A or until a visible contra-lateral forelimb movement was evoked. Movements were collected by a second experimenter that was blind to the stimulation coordinate in the grid. Fig 1 shows averaged maps of forelimb movements evoked at 20 (panel A), 40 (panel B) and 60 (panel C) *μ*A current thresholds (for details see S1 Text). In order to further validate the location of our recording electrodes, we performed optogenetic stimulation of the RFA in Thy1-ChR2 transgenic mice expressing ChR2 mainly in layer V corticospinal neurons, simultaneously recording electromyographic (EMG) activity from the contra-lateral forelimb muscle (Triceps Brachii). On Fig 1D a representative motor evoked potential (MEP) recorded from the forelimb contralateral to the stimulated RFA is reported (for additional details see S1 Text).

### Ischemic lesion and electrodes positioning

In 6 animals a photothrombotic cortical lesion in the right CFA was induced. This procedure is based on systemic infusion of the photosensitive dye Rose Bengal that, with a focal illumination through the skull, is locally activated causing radical formation, disturbance of endothelium, platelet aggregation and coagulation cascades that result in the occlusion of small vessels [37, 38].

Briefly, each mouse was anesthetized with Avertin (0.02 ml/g), placed in a stereotaxic apparatus with body temperature maintained at 37°C. Mice were then injected with Rose Bengal (Sigma-Aldrich, St. Louis, MO; 0.2 mL of a 10 mg/mL solution) intra-peritoneally. After a waiting time of 5 minutes to allow the dye to reach cerebral blood vessels, the brain was illuminated through the intact skull for 15 minutes using a cold light source (CL 6000, ZEISS, Oberkochen, Germany) connected to a 20X objective that was positioned 0.5 mm anterior and 1.75 mm lateral from bregma [39].

Immediately after ischemic phototrombotic injury, two burr holes were drilled in both hemispheres at mm from Bregma, see Fig 1E: (i) +2 anterior, +1.25 lateral. These coordinates correspond to the centre of the RFA, according to literature and to our ICMS experiments (see Fig 1A–1C and section Intra-cortical microstimulation and optogenetic experiments). An additional hole was drilled at the centre of the occipital bone to facilitate the insertion of a surgical screw that was used both as ground reference and to give more stability to the recording implant. After screw positioning, in each hemisphere an insulated tungsten electrode was stereotactically inserted in the center of the drilled holes at 700 *μ*m of depth to collect neural activity from the center of the RFA while a reference electrode was positioned in the immediate neighborhood on the overlying dura. The two bipolar electrodes were then soldered to the pins of a connector. Finally, the ground pin was connected to the occipital screw and a first layer of dental cement (Super Bond CeB, Sun Medical Co, Japan) was distributed to secure all the components to the skull surface. After the first layer of cement was dried, a second layer of cement (Paladur, Pala, Germany) was distributed to enclose all the electrical components. Glucose and Paracetamol were administered and mice were then allowed to recover from the surgery. Control animals (*n* = 6) were subjected to the same surgery procedure and received intra-peritoneal injection of Rose Bengal while skull illumination was not performed.

After all surgical procedures, animals were carefully stitched and treated with intra-operative analgesia (tramadol 10 mg/kg) and intramuscular injection of cortisone (Bentelan 0.05 ml) upon waking from anesthesia. As a further analgesic, paracetamol (100 mg/kg) were administered for 4 days post-operation in drinking water.

### LFPs acquisition

After surgery, spontaneous neuronal activity in both controls and ischemic animals was acquired during freely moving behaviour once a week for three weeks, starting from day 9. We did not acquire signals at earlier stages to allow for complete recovery from surgery and because of possible variations in LFPs due the post-stroke diaschisis and inflammation [40]. Signals from each bipolar electrode were acquired, 10000X amplified and 100Hz low-pass filtered through a 2-channels extracellular amplifier (Npi electronic, Germany) and digitized with a sampling rate *f*_*s*_ = 200 Hz through a USB DAQ board (NI USB-6212 BNC, National Instruments, USA) by means of a custom-made software developed in LabWindows CVI (National Instruments, USA).

Before recording, mice were left free to walk in the apparatus for 10 minutes to let them familiarize with the environment and allow the LFP signal to stabilize.

Twelve animals were recorded (6 stroke and 6 control) during freely moving sessions of 30 minutes. All animals were recorded up to 23 days post surgery. Each signal consisted of a bivariate time series representing the LFPs simultaneously recorded at sampling interval Δ*t*: = 1/*f*_*s*_ = 5 *ms* from ipsi-lesional (*X*^ipsi^(*t*_*i*_), *t*_*i*_ = 0, Δ*t*, …, (*N* − 1)Δ*t*, *N* = 3.6 × 10^5^) and contra-lesional hemisphere (*X*^contra^(*t*_*i*_), *t*_*i*_ = 0, Δ*t*, …, (*N* − 1)Δ*t*, *N* = 3.6 × 10^5^). Signals were then band-pass filtered in (0.5 − 50)Hz.

### Preprocessing: Removal of recording artifacts

The quality of the LFP recordings highly depends on the stability of the electrodes and on the movements of the animal [27]. In our experiments, we observed the occurrence of a high amplitude deviations of the signal whenever the animals bumped into the wall of the apparatus or jumped with a 180° turn, as already shown in previous works [27].

**Fig 2:**
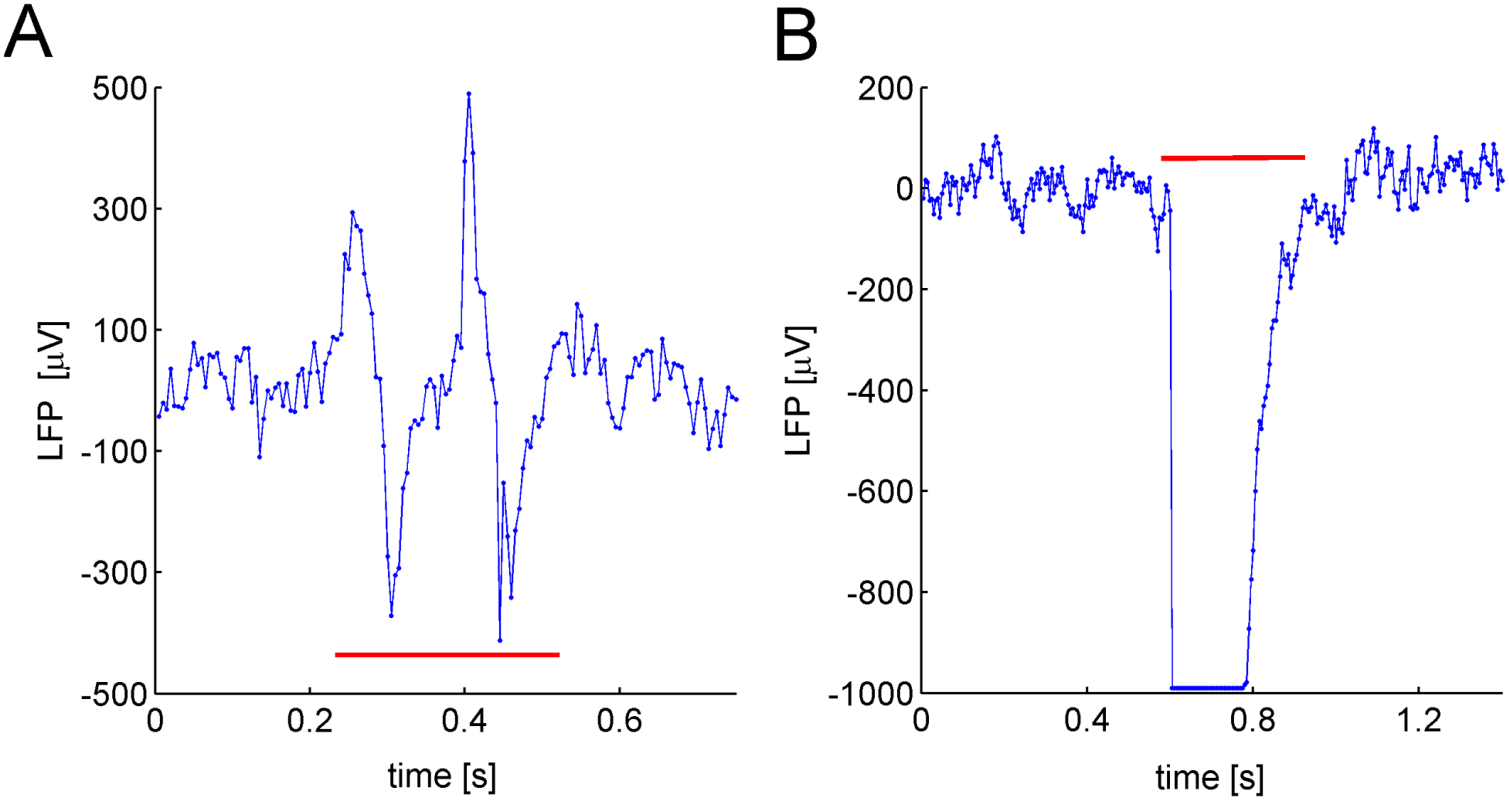
LFP Artifacts. A) and B) Two examples of typical recording artifacts, both indicated by the horizontal red line. Each artifact is characterized by a large deviation of the signal from its mean value.

An experienced experimenter visually identified the part of signals corresponding to such events. We quantified that the amplitude of the artifacts, *A*, ranged in [−995.6, 556.2]*μ*V, whereas the duration, *D*, fell in the interval [0.035, 1.095]s. Two examples of artifacts are shown in Fig 2.

**Fig 3:**
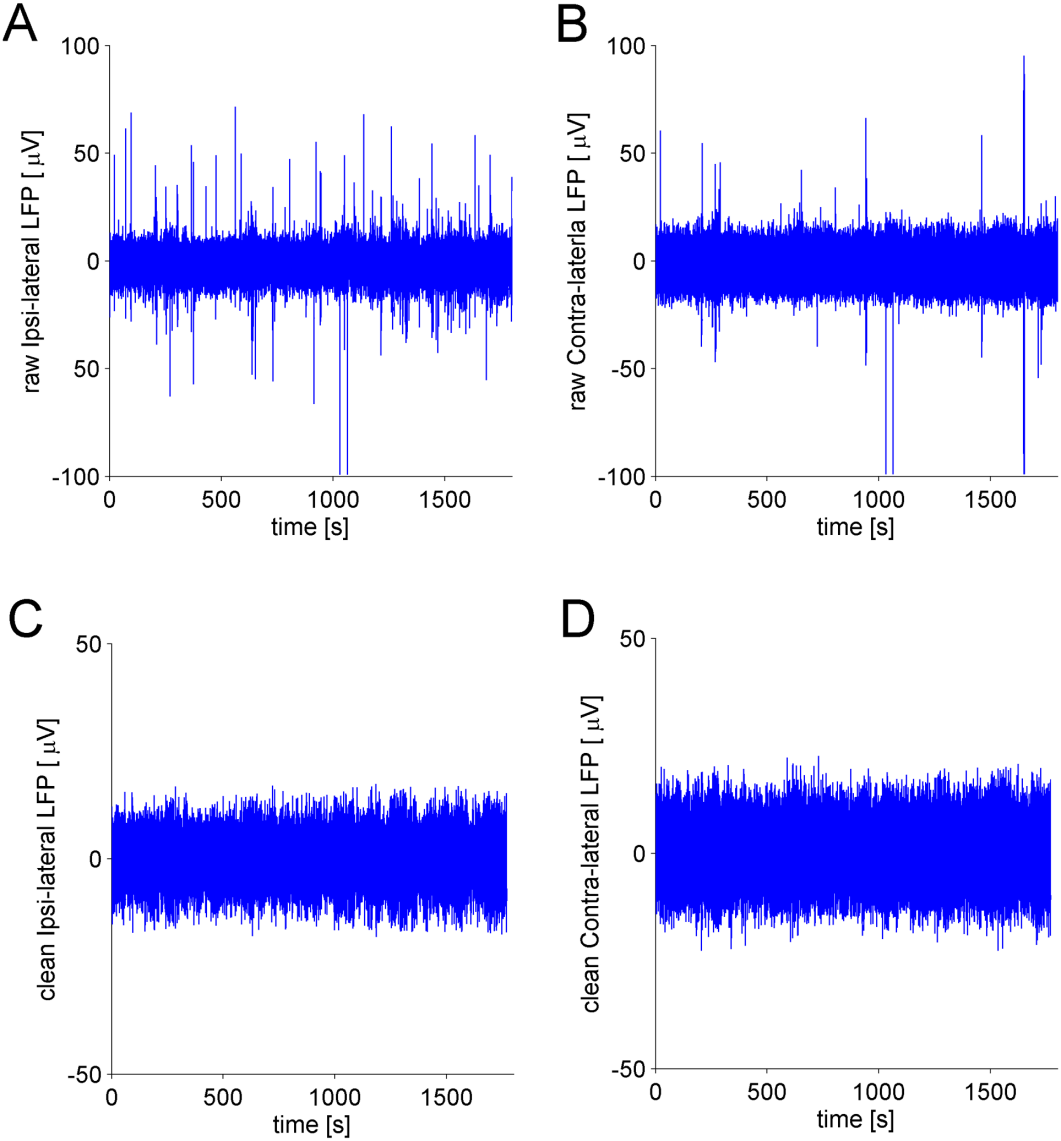
Raw and Clean LFPs. A) The raw ipsi-lateral LFP signal recorded from a single animal shows several artifacts, i.e. large deviations from the average of the signal. B) Same as A) but for the contra-lateral LFP signal. C) The cleaned ipsi-lateral LFP signal after the application of the artifact removal algorithm. D) Same as C) but for the contra-lateral LFP signal.

Based on this characterization, we implemented an algorithm to automatically remove the artifacts. Briefly, the algorithm processed the ipsi-lateral and contra-lateral signals simultaneously. We set a threshold value to identify high amplitude deviations of the signals, i.e. peaks. Each peak was characterized by means of its starting and ending time points, which allowed for defining the temporal interval of the corresponding artifact. After this identification, the artifact was removed from the signal. Finally, the two parts of the signals, present at the sides of the removed artifact, were linked by using a “joining” procedure (for a detailed explanation of the whole removal procedure, see S2 Text and S2 Fig). The Fig 3 shows cleaned LFPs after the removal procedure performed by the algorithm on raw data.

Before applying the algorithm to LFPs data, we tested it on artificial models where analytical expression of quantities of our interest (power band, cross correlation, mutual information, Granger causality) were known. On these signals, generated with artificial models, we then added a number of artifacts similar to that typically identified in our recordings. Finally, we used our algorithm on these contaminated data sets and demonstrated that: i) the presence of artifacts produce distorted estimation of the considered measures, ii) after data cleaning the true value of these measures is restored (see S3 Text and S2 Fig).

### Spectral Analysis

The power spectrum of the signals was estimated by using the Welch method of averaging modified periodograms [41, 42]. We focused on estimating the power content of the standard neurophysiological spectral bands: *δ* = (0.5 − 4)Hz, *θ* = (4 − 8) Hz, *α* = (8 − 12) Hz, *β* = (12 − 30) Hz, *γ* = (30 − 50) Hz of the LFP signal [43].

In particular, we considered the relative power of a generic *λ* = *δ*, *θ*, *α*, *β*, *γ* spectral band with respect to the total power of the signal as follows

$$\begin{array}{c} P_{\text{rel}}\left(\lambda \right)=\frac{\sum_{f_{k}\in \lambda }P\left(f_{k}\right)}{P_{\text{tot}}} \end{array}$$ (1)

where *f*_*k*_ is a frequency belonging to the *λ* spectral band, *P*(*f*_*k*_) is the corresponding power and *P*_tot_ is the total power of the LFP signal.

### Cross Correlation

To quantify the linear dependence between the LFP signals sampled at *t*_*i*_ = 0, Δ*t*, …, (*N* − 1)Δ*t* from ipsi-lateral (*x*(*t*_*i*_)) and contra-lateral (*y*(*t*_*i*_)) hemispheres, we used the cross correlation coefficient [41, 42] defined as

$$\begin{array}{c} \rho_{xy}:=\frac{1}{N}\frac{\sum_{i=0}^{N-1}\left(x\left(t_{i}\right)-\bar{x}\right)\left(y\left(t_{i}\right)-\bar{y}\right)}{\sigma_{x}\sigma_{y}} \end{array}$$ (2)

where $\bar{x}\left(\bar{y}\right),\sigma_{x}\left(\sigma_{y}\right)$ are the mean and the standard deviation of the LFP signal, respectively and *N* = 3.6 × 10^5^ is the total sample size of the two signals. In order to avoid possible bias due to the presence of nonstationarities, a windowing procedure of the signals was adopted. Thus, the signal was divided into *N*_*w*_ windows, each containing *N*_*p*_ data points (*N*_*p*_ = 4096 see section Reduction of inter-hemispheric correlation after stroke), and the mean value *ρ*_*xy*_ was obtained as

$$\begin{array}{c} \bar{\rho }=\frac{1}{N_{w}}\sum_{l=1}^{N_{w}}\rho_{l} \end{array}$$ (3)

where *ρ*_*l*_ (*l* = 1, 2, …, *N*_*w*_) is the value of the cross correlation coefficient on the *l*-th window.

### Mutual Information

To quantify the nonlinear correlations between LFPs recorded from the ipsi-lateral and contra-lateral hemispheres the mutual information (MI) was used [41, 42]. A method to estimate the MI between the two LFP signals, *x*(*t*_*i*_) and *y*(*t*_*i*_) (*t*_*i*_ = 0, Δ*t*, …, (*N* − 1)Δ*t*, *N* = 3.6 × 10^5^), is that of partitioning their supports (*X*, *Y*) in a number of bins *N*_*b*_ leading to the following formula:

$$\begin{array}{c} I_{XY}:=\sum_{ij}p_{XY}\left(i,j\right)\log\frac{p_{XY}\left(i,j\right)}{p_{X}\left(i\right)p_{Y}\left(j\right)}, \end{array}$$ (4)

where *p*_*X*_(*i*) (*p*_*Y*_(*j*)) is the probability to find the value of the random variable *X* (*Y*) in the *i*-th (*j*-th) bin (*i*, *j* = 1, …, *N*_*b*_) and *p*_*XY*_(*i*, *j*) is the corresponding joint probability [41, 42]. We adopted the procedure of data windowing as in the case of the cross correlation to estimate the mean value of MI (*N*_*p*_ = 4096 and *N*_*b*_ = 16, see sections Cross Correlation and Reduction of inter-hemispheric correlation after stroke).

### Granger Causality

The Granger causality method was used to assess whether the ipsi-lateral signal (*x*(*t*_*i*_)) was useful in predicting the contra-lateral one (*y*(*t*_*i*_)) and vice-versa, ascertaining the coupling directionality [44]. The signals were windowed through *N* = 512 points, corresponding to 2.56 s. The total strengths of the influence from *y* → *x* and from *x* → *y* were quantified by using two coefficients $G_{y\to x}^{p}$ and $G_{x\to y}^{p}$ respectively, computed over the windows of the signals (see definitions in S4 Text). We used these quantities to define a coefficient quantifying the hemispheric dominance:

$$\begin{array}{c} G^{\text{HD}}:=\frac{G_{y\to x}^{p}-G_{x\to y}^{p}}{G_{y\to x}^{p}+G_{x\to y}^{p}}. \end{array}$$ (5)

Indeed, a value of *G*^*HD*^ = 0 implies that the interactions between the two hemispheres are balanced (ipsi-lateral→contra-lateral = contra-lateral→ipsi-lateral), while values *G*^*HD*^ > 0 and *G*^*HD*^ < 0 indicate that a contra-lateral→ipsi-lateral and ipsi-lateral→contra-lateral dominances are present, respectively.

Furthermore, the symmetry level of the inter-hemispheric interaction was estimated by

$$\begin{array}{c} G^{S}:=\left|G^{\text{HD}}\right|. \end{array}$$ (6)

The value of $G^{S}=0$ indicates that the coupling between the two hemispheres is symmetric, while a value $G^{S}>0$ that an unbalanced interaction is present.

Finally, we investigated the temporal dynamics of the coupling directionality. We defined the frequency distributions of hemispheric dominance, *f*_*HD*_ counting the number of time windows in which ipsi − lateral → contra − lateral *dominance*, contra − lateral → ipsi − lateral *dominance* or *balance* occurred, and then normalized by the total number of windows (see definitions in S4 Text).

### Numerical Implementation and Statistical Analysis

The numerical codes used to estimate the different measures (cross correlation, MI, spectral bands, $G^{S}$) were written in Matlab (Matworks, USA). The spectral bands and cross correlation coefficient were computed by using the Signal Processing Toolbox. The MI was estimated by using a function provided by the CRP toolbox [45], whereas the $G^{S}$ index was calculated by employing available functions of the MVGC toolbox [46].

To assess whether two or more quantities were statistically different, we used a two-way repeated measures Anova provided by the software SigmaPlot (Systat Software, USA).

## Results

In the following sections we describe the results obtained from the analysis of LFPs recorded from both hemispheres of either naïve or ischemic mice. Ischemia was induced unilaterally (right hemisphere) in the CFA, and bipolar electrodes were positioned in both RFAs (Fig 1E). Coordinates for lesion and electrode implantation were based on intracortical microstimulation mapping experiments (see Fig 1A–1C) and LFP recordings were collected from freely moving mice (at 9, 16 and 23 days post-implantation).

### Post-stroke changes in LFP power spectra

Spectral analysis (see section Spectral Analysis) was used to calculate the distribution of the relative powers over the bands: *δ* = (0.5 − 4)Hz, *θ* = (4 − 8) Hz, *α* = (8 − 12) Hz, *β* = (12 − 30) Hz, *γ* = (30 − 50) Hz for stroke and naïve mice.

**Fig 4:**
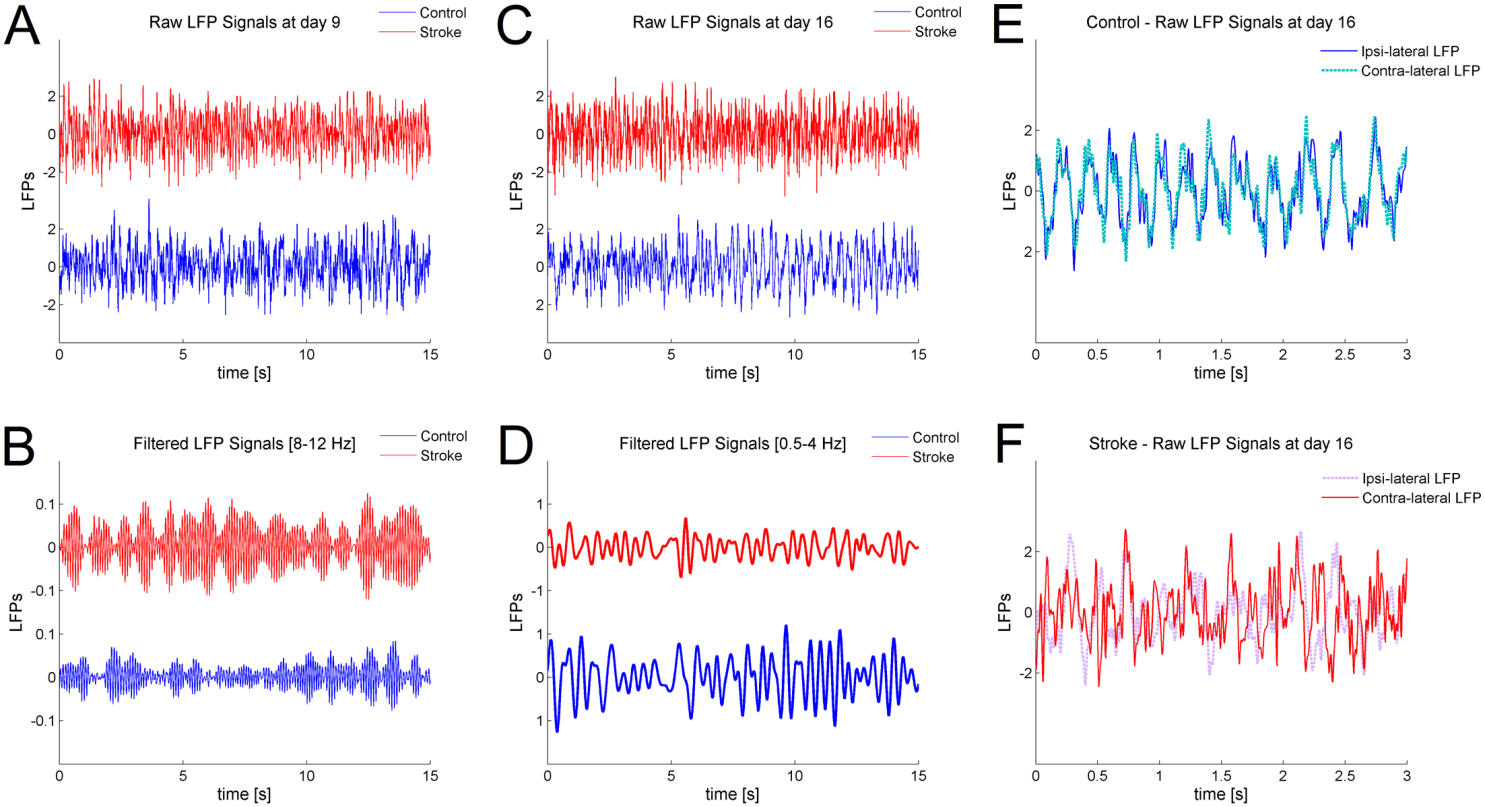
Representative differences in LFPs after stroke on frequency oscillations and correlation. A) A window of 15 seconds of two raw signals recorded at day 9 in control and stroke animals, red and blue trace respectively, is shown. B) The same signals after pass band filtering between 8 − 12 Hz, corresponding to *α* frequency, is shown. The power of the signal from the stroke animal (red) is higher compared to the control (blue), in agreement with the result shown in Fig 5A and 5B. C) A window of 15 seconds of two raw signals recorded at day 16 in control and stroke animals, red and blue trace respectively, is shown. D) The same signals after pass band filtering between 0.5 − 4 Hz, corresponding to *δ* frequency, are shown. In this case, the power of the stroke signal (red) is lower compared to the control (blue), in agreement with the result in Fig 5C. E) and F) A window of 3 seconds from the two raw LFPs, simultaneously recorded in the ipsi-lateral and contra-lateral RFA of a control (Panel E) and stroke (Panel F) animals at day 16. The two traces of the control animal show a high correlation, as reported in Fig 6A and 6B. On the other hand, the signals recorded simultaneously from the two RFAs of the stroke animal show a lower correlation compared with the control case, as reported in Fig 6A and 6B.

**Fig 5:**
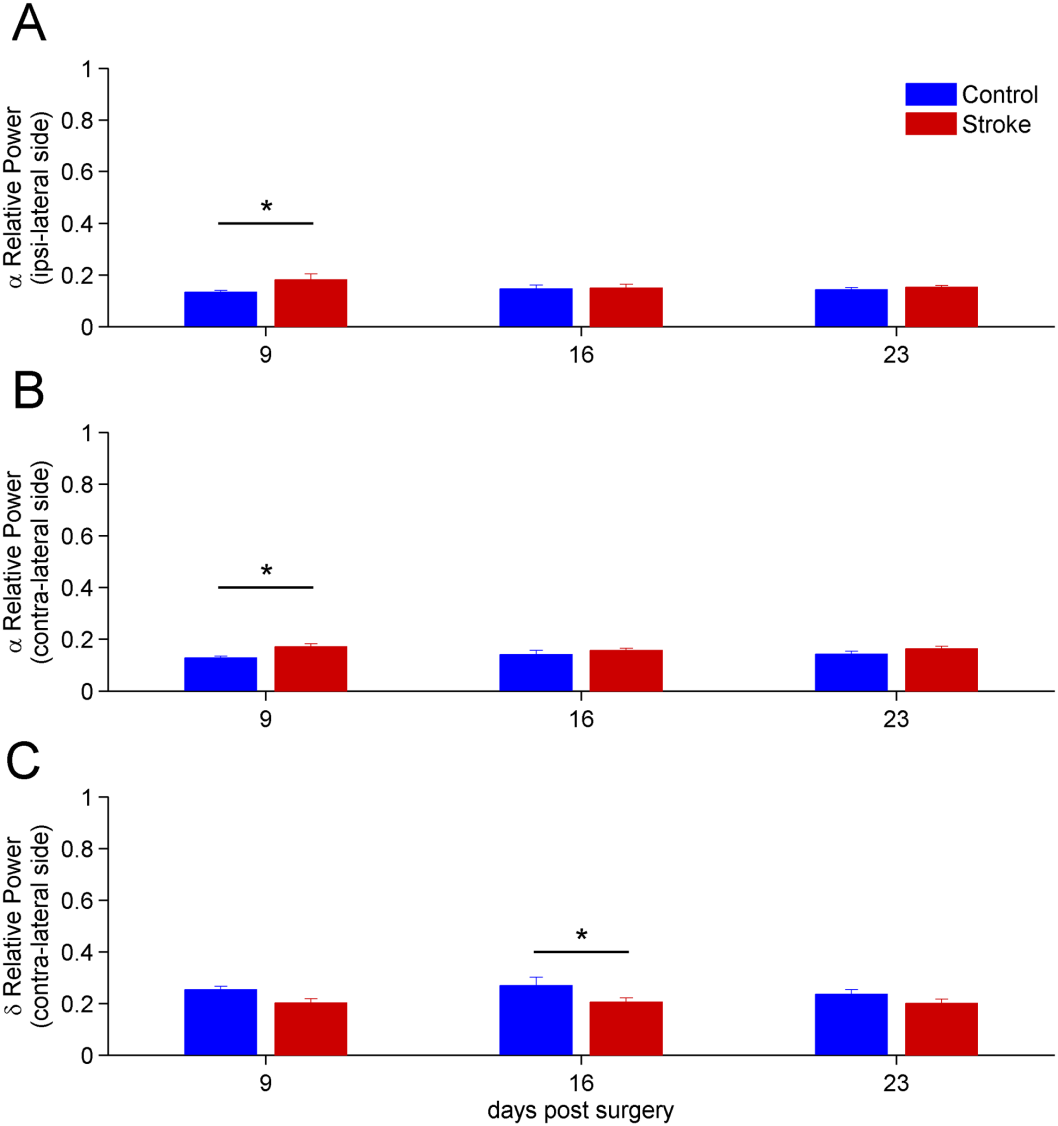
Time evolution after stroke of the mean relative powers of *α* and *δ* spectral bands. Only bands showing significant differences in stroke vs. naïve animals are shown. A) Values of the relative power of the *α* = (8 − 12) Hz band for the ipsi-lesional are shown. The result for day 9 shows significant statistical differences between stroke and control (*p* < 0.05). B) Values of the relative power *α* = (8 − 12) Hz band for the contra-lateral hemisphere is shown. As in the case of the ipsi-lesional hemisphere, significant statistical differences were found at day 9 (*p* < 0.05). C) Significant reduction of the *δ* band in the contralesional hemisphere, 16 days after stroke. For all panels the error bars represent the corresponding standard errors.

We found no significant longitudinal changes in the LFP power spectra of naïve animals, indicating stability of the recordings over time. A two-way repeated measures Anova revealed that small but consistent differences could be detected in stroke vs. naïve mice, restricted to the *α* and *δ* bands. Representative differences in LFPs between control and stroke mice are shown on Fig 4A–4D. Specifically, the power of the *α* band significantly increased at day 9 (*p* < 0.05), both in the ipsi-lesional and contra-lesional hemisphere of stroke mice (Fig 5A and 5B). No significant changes were found at later time points, apart from a reduction in the power of the *δ* band for the contra-lesional hemisphere at day 16 (*p* < 0.05, see Fig 5C). The high frequency parts of the spectrum, i.e. *β* and *γ* band, showed no variations in stroke vs. control animals (data not shown).

### Reduction of inter-hemispheric correlation after stroke

**Fig 6:**
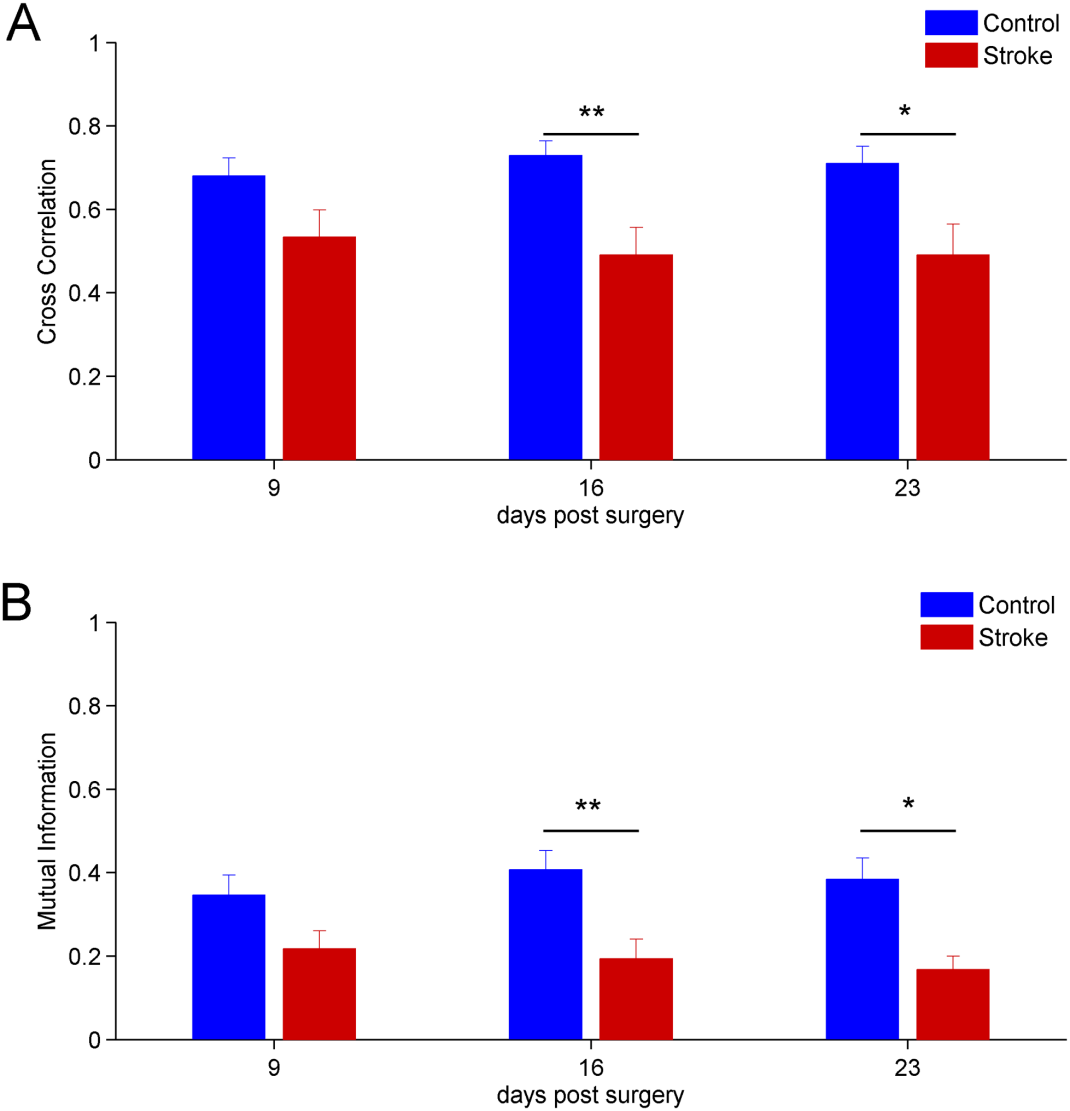
Time evolution after stroke of the mean linear and non-linear inter-hemispheric correlation measures. A) Cross correlation between the ipsi-lateral and contra-lateral hemisphere. Days 16 and 23 show significant statistical differences, *p* < 0.01 and *p* < 0.05, respectively. B) Longitudinal evolution of mutual information. Statistically significant differences between stroke and controls are present at days 16 (*p* < 0.01) and 23 (*p* < 0.05). For all panels the error bars represent the corresponding standard errors.

We used the linear measure cross correlation (see section Cross Correlation) to estimate the level of functional coupling between the two hemispheres. In Fig 6A the mean values of cross correlation of LFP signals and standard errors, for the stroke and control groups, are shown. We calculated the cross correlation value through data windowing with *N*_*p*_ = 4096 (see section Cross Correlation)and found significant statistical differences between the control and the stroke group at day 16 (*p* < 0.01) and 23 (*p* < 0.05) after surgery (two-way repeated measures Anova). Specifically, the mean value of the cross-correlation was dampened in ischemic animals. Representative differences in correlation between LFPs recorded in control and stroke mice are shown on Fig 4E and 4F.

The results of the mutual information analysis were consistent with the case of cross correlation (using the same data windowing). In Fig 6B, the mean values of mutual information (number of bins *N*_*b*_ = 16, see section Mutual Information) and corresponding standard errors are shown. No statistical differences were found at day 9 post surgery. Instead, significant statistical differences between the control and the stroke group were found at day 16 (*p* < 0.01) and 23 (*p* < 0.05) after surgery (two-way repeated measures Anova). We also verified the robustness of this results by changing either the number of points in each window or the number of bins (data not shown).

Moreover, by using random and independent shuffled surrogate data, we found that the observed reduced inter-hemispheric correlation values were above chance level. Indeed, after shuffling operation, the statistical comparisons of the value of cross correlation and mutual information between groups (stroke vs. control) showed no significative differences, indicating that the decrease of inter-hemispheric correlation was not due to the chance (data not shown).

Altogether, the results obtained with the cross correlation and mutual information analysis suggest that reduction of inter-hemispheric functional connectivity between RFAs occurs following stroke.

#### Early reduction of inter-hemispheric correlation on spectral bands

Since we found post-stroke alterations in inter-hemispheric correlations on the whole LFP signals, we then investigated the linear and non-linear correlations on signals with components belonging to a specific band *λ* (*δ*, *θ*, *α*, *β*, *γ*). Therefore, the signals were filtered to select the specific band components and linear (i.e. cross correlation) and non-linear (i.e. mutual information) correlations were then computed (see Cross Correlation and Mutual Information sections).

**Fig 7:**
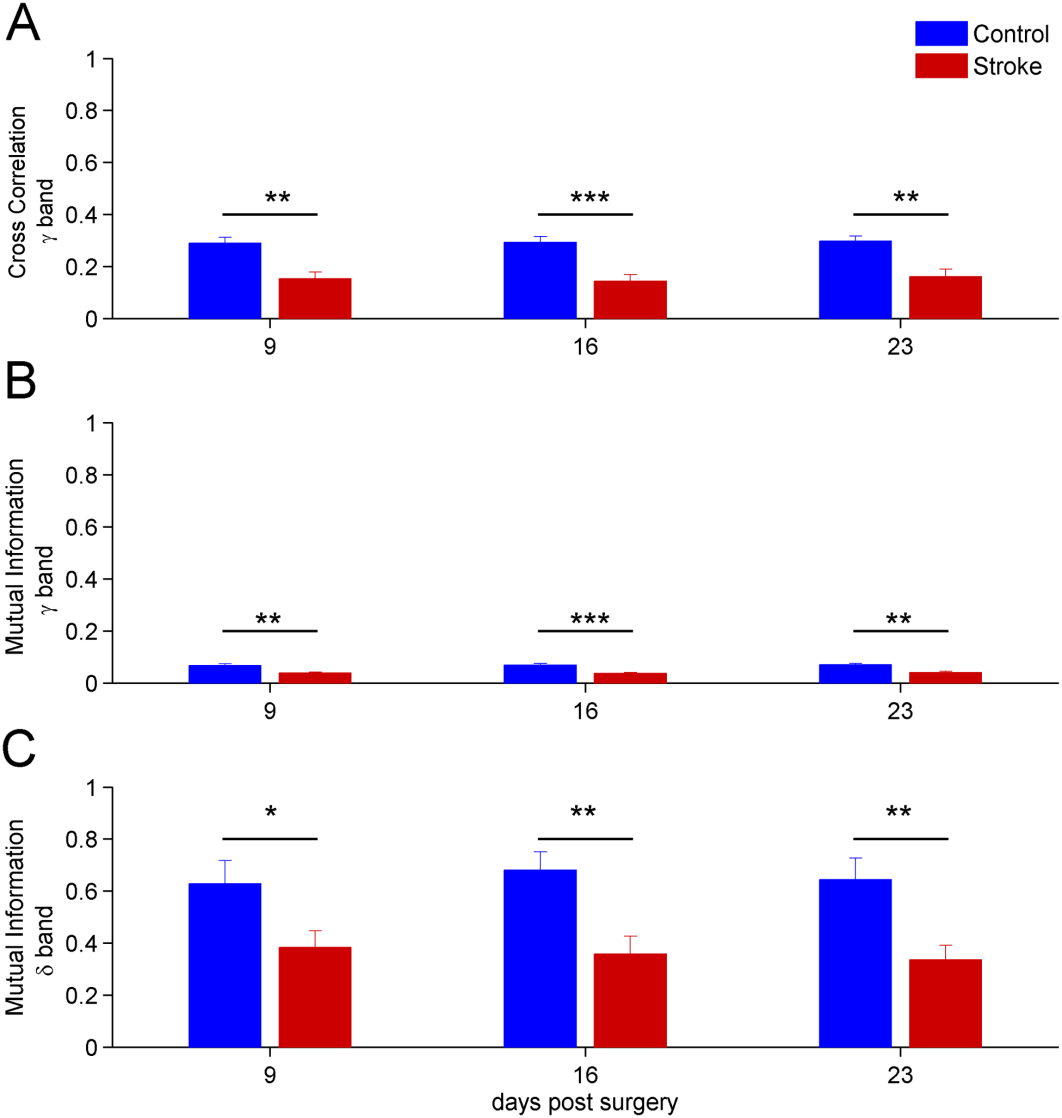
Time evolution after stroke of the inter-hemispheric correlation on *δ* = (0.5 − 4) Hz and *γ* = (30 − 50) Hz bands. A) Cross correlation between the ipsi-lateral and contra-lateral hemisphere for signals having frequencies on *γ* = (30 − 50) Hz band. The differences are highly significant at day 16 *p* < 0.001. B) Same as A) but using the mutual information measure. C) Values of the mutual information on *δ* = (0.5 − 4) Hz band. Note the significant reduction in stroke mice, already apparent at day 9. For all panels the error bars represent the corresponding standard errors.

At day 9, a significant stroke-dependent reduction in inter-hemispheric cross-correlation and mutual information for *γ* band occured (two-way repeated measures Anova, *p* < 0.01, see Fig 7A and 7B). Moreover, the values of the mutual information for *δ* band also indicated a reduction in stroke mice at day 9 (Fig 7C). Thus, changes in inter-hemispheric coupling for the *γ* and *δ* range preceded the variations observed using the whole LFP signal (see Fig 6). Notably, the *γ* band manifested highly significant differences (two-way repeated measures Anova, *p* < 0.001) at day 16 (Fig 7A and 7B). The results found in the other bands are in agreement with the findings of the analysis on the whole signals, i.e. they show a reduction in inter-hemispheric correlation at days 16-23 post-stroke (data not shown).

The same analysis was also performed on surrogate time series and the results indicated that the decrease of inter-hemispheric correlation was above chance level (data not shown).

### Unbalanced inter-hemispheric interaction and hemispheric dominance fluctuations following stroke

We used the Granger causality test to determine the causal influences between the two forelimb Pre-Motor Areas in the two hemispheres. In particular, we estimated the *G*^*HD*^ and $G^{S}$ coefficients to investigate hemispheric dominance and symmetry of interaction, respectively (see for definitions Eqs (5) and (6) in section Granger Causality and S4 Text).

**Fig 8:**
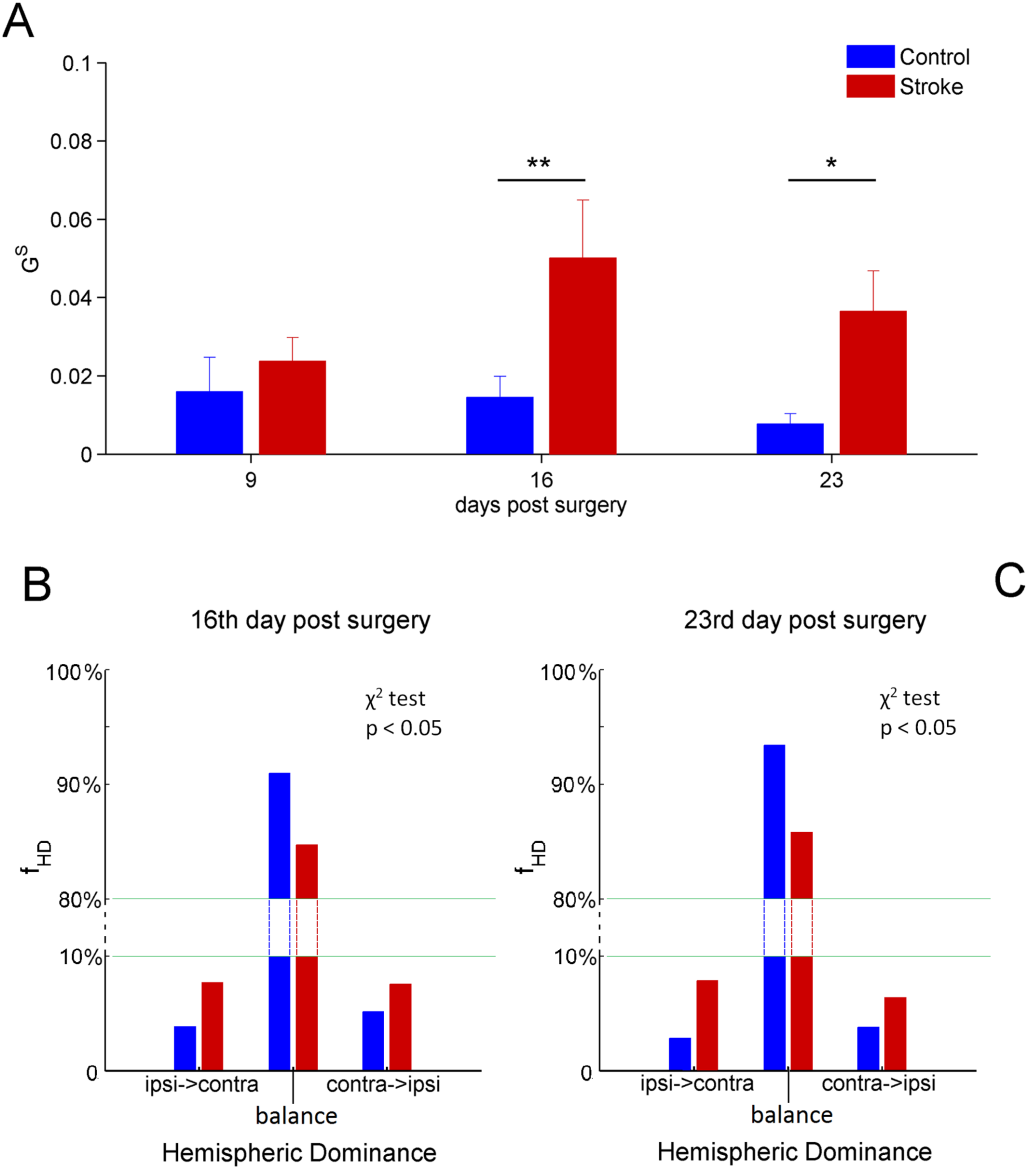
Mean values of $G^{S}$ and frequency of hemispheric dominance *f*_*HD*_. A) Level of symmetry of the inter-hemispheric interaction evaluated through the values of $G^{S}$. The first week does not present significant statistical differences. On the other hand, days 16 and 23 highlight significant statistical differences *p* < 0.01 and *p* < 0.05, respectively (two-way repeated measures Anova). B) Frequency of hemispheric dominance *f*_*HD*_ (expressed in percentage) at days 16 post surgery. The distributions are zoomed in the ranges 0 − 10% and 80 − 100% to magnify the variation of *f*_*HD*_. The frequency distributions of stroke and control mice are significantly different (*p* < 0.05 *χ*^2^-test). C) Same as B) but for 23 post surgery. Also in this case the frequency distributions are significantly different between stroke and control mice (*p* < 0.05 *χ*^2^-test). For the top panel the error bars represent the corresponding standard errors.

To calculate the values of Granger causalities $G_{\text{ipsi}\to \text{contra}}^{p}$ and $G_{\text{contra}\to \text{ipsi}}^{p}$ (see S4 Text) we adopted a data windowing with *N* = 512 data points, corresponding to 2.56s, and an autoregressive models of order *p* = 15 (see S4 Text). The Fig 8A shows the time evolution of the mean values of $G^{S}$ following stroke. Similarly to the inter-hemispheric correlation, we did not find significant differences between stroke and control groups at day 9 post surgery, whereas differences at 16 (*p* < 0.01) and 23 (*p* < 0.05) days were observed (two-way repeated measures Anova). Specifically, $G^{S}$ was higher in the stroke group, indicating a more unbalanced coupling between the two RFAs.

However, this result cannot be explained by a net overall driving from one of the two hemispheres. Indeed, single animals showed opposite hemispheric dominance ($G_{\text{ipsi}\to \text{contra}}^{p}>G_{\text{contra}\to \text{ipsi}}^{p}$ or vice-versa) even inside the same group (data not shown). For this reason, the *G*^*HD*^ parameter, accounting for the mean hemispheric dominance, did not show differences between stroke and control mice at each time point post surgery (two-way repeated measures Anova, data not shown).

Moreover, we investigated the temporal dynamics of the coupling both in stroke and control groups during the freely moving recordings. Indeed, we examined for each temporal window whether the signal in the right RFA (ipsi-lesional) was effective in influencing the signal in the left RFA (contra-lesional) or vice-versa.

Thus, we constructed the frequency distributions of hemispheric dominance, *f*_HD_, for both stroke and control mice. The *f*_*HD*_ distributions are shown in Fig 8B and 8C. We found that the distributions for stroke and control groups were significantly different (*p* < 0.05; *χ*^2^-test). In particular, stroke mice showed an increased number of windows with ipsi-lesional and contra-lesional dominance (≈10% each one, see Fig 8B and 8C), indicating that hemispheric dominance fluctuates more frequently after a cortical infarct.

## Discussion

**Fig 9:**
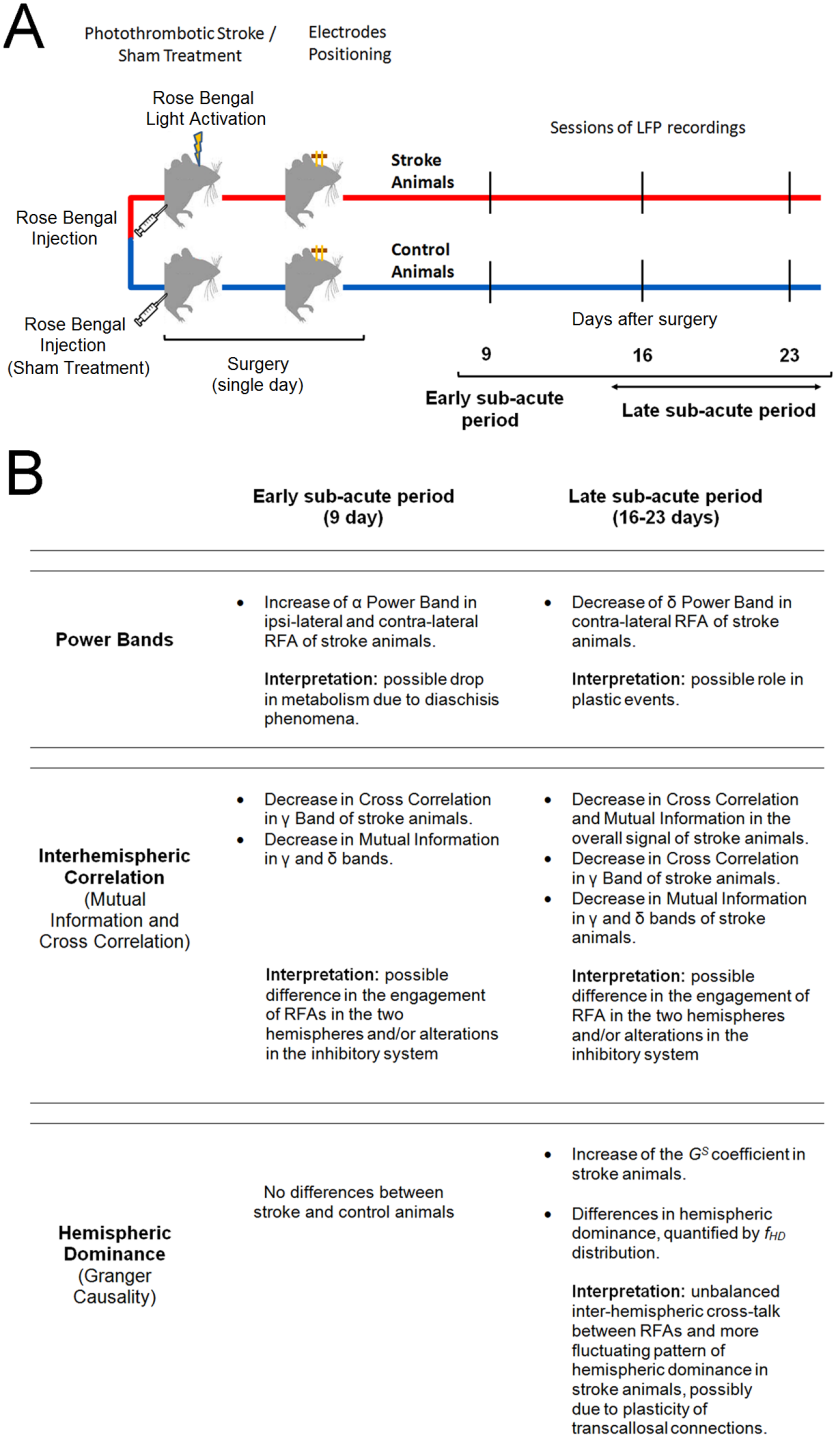
Schematic of the experimental protocol and summary of the results and possible interpretations. A) The experimental layout is described in all of its parts: the stroke induction in CFA (or sham treatment) was followed by the positioning of the bipolar electrodes into the two RFAs. The LFPs were then recorded 9,16 and 23 days after surgery in the freely moving animals. B) The overall results are presented following the proposed separation in an early (day 9) and a late sub-acute period (days 16 and 23). The possible biological interpretation is also reported.

In this study we investigated longitudinal alterations in intra-hemispheric neural activity and inter-hemispheric coupling in Pre-Motor Areas of the mouse cortex after stroke in freely behaving mice. A schematic of the experimental protocol is shown on Fig 9A. This condition was chosen to study spontaneous activity reflecting a self-determined behavior. Reciprocal connections between Pre-Motor, RFA, and Primary Motor Cortex, CFA, have been anatomically identified in rodents [18]. Thus, an injury in the CFA might influence the neural circuitry of ipsi-lateral RFA, inducing an alteration in inter-hemispheric interactions with contra-lateral homotopic RFA. We found significant differences in several statistical coefficients (power band, cross correlation, mutual information, $G^{S}$, frequency of hemispheric dominance *f*_*HD*_) indicating alterations of both local cortical activity and interactions between homologous RFAs after stroke. A summary of the overall results is presented on Fig 9B.

We found that decreased correlation between hemispheres in *γ* and *δ* bands, as well as increased power of *α* band, are early indicators (9 day) of alterations following stroke. At later stages (16 and 23 days), causal interactions between hemispheres are modified.

We separately discuss the cortical reorganization observed at day 9 (early sub-acute period) and that observed 16-23 days after surgery (late sub-acute period). We chose these time points since they roughly correspond to the first 3 months post-stroke in humans, i.e. the phase of highest circuit plasticity [47]. This correspondence between human and rodent post-stroke stages can be determined based on (i) longitudinal changes in fMRI motor system activity and (ii) length of the post-stroke “critical period” for recovery. In human strokes, enhanced activity in the contra-lesional hemisphere is observed early after injury (about 10 days), followed by a relative increase of activity in ipsi-lateral areas adjacent to the stroke at 3-6 months [48, 49]. Similarly, in rodents at early stages (3 days) after stroke, extensive activation-induced responses is detected in the contra-lesional hemisphere. After 14 days, reduced involvement of the contra-lesional hemisphere and significant responses in the infarction periphery are reported. Furthermore, almost all recovery from impairments occurs during a post-stroke sensitive period, extending up to about 3 months in humans and one month in rodent models (reviewed in [47]). Altogether, these data indicate that the time points sampled in the present study (9-16-23 days) lie within the critical period for post-stroke plasticity and correspond to approximately 1 month to 3 months post-stroke in humans (early sub-acute and late sub-acute period, respectively).

### Early sub-acute period

In the early sub-acute period, an increase of the *α* band power was observed in both the hemispheres. The functional meaning and the mechanisms underlying the generation of the *α* band activity are still mostly unknown. *α* oscillations are thalamocortical rhythms occurring in relaxed but still waking brain states and it could be divided in different patterns: generic *α*, *μ* rhythm and *τ* rhythm. Each of them is associated to different brain states but all *α* rhythms share a condition of absence of stimuli, movements or any brain input. In particular, in rodents, that present a highly extended whisker representation, the dominant oscillation is the *μ* rhythm being highly related to the somatosensory system during non-working state [50, 51]. Interestingly, in humans high oscillatory *α* activity has been associated with low brain metabolism [52], so we can argue that the early *α* power increase could be due to a drop in brain metabolism for diaschisis phenomena.

Moreover, in the first period we also observed a reduction in cross correlation in *γ* band and mutual information in *γ* and *δ* bands. This could suggest a different engagement of PMAs in stroke animals during the early sub-acute period. Joho et al. [53], demonstrated that fast *γ* and slow *δ* oscillations in somatomotor cortex are altered in freely behaving mutant mice devoid of a potassium channel expressed by fast-spiking Parvalbumin inhibitory interneurons thus indicating a role of such cells in regulation of rhythmic brain activity [53]. Indeed, an altered firing of inhibitory interneurons in the ipsi-lesional cortex could be the cause of a different modulation of cortical activity in comparison to the contra-lateral hemisphere, leading to a reduction of the inter-hemispheric statistical correlation. Our results confirm a key role of *γ* oscillations in cross-talking between brain regions [54], especially in combination with slower frequencies [54, 55].

### Late sub-acute period

In the late sub-acute period, we observed a decreased *δ* power in the contra-lesional hemisphere. Moreover the cross correlation and the mutual information of stroke mice, calculated both over the whole signal and over the different frequency bands, showed a significant reduction with respect to controls. In addition, an increase of the $G^{S}$ coefficient, indicating a reduction of the symmetry in the causal inter-hemispheric cross-talk, was also found.

*δ* waves are another thalamocortical activity, observed in drowsy or sleeping animals [56] which seems to be related to a cholinergic activation [50]- They may have a role in memory consolidation [57] and prepare the animal in resting state to receive inputs [58]. *δ* band in humans is associated with deep sleep but also with learning and motivation [59]. A recent study of intermittent theta-burst stimulation in M1 of healthy humans showed that induction of long-term potentiation phenomena is coupled with an increase of *δ* wave activity over the ipsi-lateral frontal cortex [60]. These findings suggest a role of *δ* activity in plastic events during wakefulness. Pathological *δ* activity has been often outlined by EEG studies in humans after cerebral lesions. In particular, it has been found that patients with cortical strokes could exhibit increased *δ*-activity in perilesional areas both in acute and in chronic stage but its functional meaning remains poorly understood [61].

Concerning inter-hemispheric interactions, our results support the idea of a robust rearrangement of transcallosal functional connectivity during the late sub-acute period following stroke. First, we noted a decrease in the coupling of the two hemispheres as indicated by cross-correlation and mutual information analysis. These data are consistent with resting-state functional magnetic resonance imaging in humans [62] and rats [63]. These studies reported a significant loss of functional connectivity between the two hemispheres following stroke that slowly recovered in parallel with spontaneous behavioural improvements.

Second, we used Granger analysis to further dissect whether the patterns of hemispheric dominance are modified after stroke. In naïve freely moving mice, we found a similar influence of each hemisphere on the other during most of the temporal windows analysed. These data are in agreement with previous works showing that in healthy freely behaving rats, no significant differences in terms of regional cerebral glucose utilization are detectable between the two hemispheres [64]. Moreover, in mice exploring a novel open-field, there was no significant bias in stronger 2-Deoxy-Glucose signal between the two hemispheres [65]. It has been demonstrated a robust relationship between changes in tissue oxygen and synaptic activity measured by LFPs [66]. These findings suggest the presence of a balanced cross-talk between the two hemispheres that reflects homeostatic mechanisms of cortico-cortical activity regulation. In contrast, in stroke mice we found an unbalanced cross-talk and a more fluctuating pattern of hemispheric dominance, with a greater proportion of temporal windows in which the influence of one RFA was significantly stronger than the effect of the other RFA. Thus, stroke caused no net changes in hemispheric dominance, but rather a less controlled balance between forelimb Pre-Motor Areas. This finding could be explained by a decrease in GABAergic markers in peri-infarct areas [67] and contra-lateral hemisphere [68]. Indeed, local activities in the two RFAs are expected to fluctuate with lower correlation level and with less interdependence under conditions of reduced inhibition.

### Possible implications for functional recovery

Previous studies have pointed-out a key role of Pre-Motor Areas in motor recovery after stroke both in clinical and preclinical studies [9, 11, 17, 69, 70]. Moreover, previous data have demonstrated a correlation between post-stroke loss of sensorimotor function and deterioration of inter-hemispheric functional connectivity in patients and animals [63, 71]. For example, in clinical studies on stroke patients, changes in *α*-band functional connectivity, both in the peri-lesional and contra-lesional cortex, have been related to improvements of functional outcomes of upper extremities [72]. Consistently, data collected in our laboratory about spontaneous evolution of post-stroke forelimb motor function, measured with skilled reaching test, showed that, despite long-lasting impairments in forelimb kinematics, the success rate in the test spontaneously recovered starting from 16 days post lesion [39]. The present findings suggest possible adaptive inter-hemispheric circuit rearrangement in Pre-Motor Areas which could be the neurophysiological substrate for spontaneous recovery of forelimb motor performance in skilled tasks.

In future studies it will be interesting to examine whether changes in inter-hemispheric coupling can be impacted by rehabilitative and plasticizing treatments [73].

### Limitations of the study

The photothrombotic mouse model used in this study reproduces ischemic insults in strictly controlled experimental conditions that cannot reproduce the complexity and heterogeneity of a human stroke. However, although in humans the extent and localization of the damage can be highly variable, strokes are mostly small in size [23], ranging from 4.5 to 14% of the ipsi-lateral hemisphere. In this context, the photothrombotic model of stroke offers standardized lesions that are well suited for quantitative analyses and investigations about connectivity and plastic phenomena after brain injury, as amply demonstrated in the literature [5, 74].

The aim of the present study was to assess electrophysiological alterations in forelimb Pre-Motor Areas (i.e. RFAs). We performed these recordings in a freely moving condition, where mice can behave with the lowest constriction thus reflecting a self-determined behavior [30]. This setup is largely used in literature not only for studies about stroke [75] but also for different fields of investigations involving memory [32], sensorimotor [31] and visual system [33].

We employed bipolar electrodes placed intracortically in the center of the RFAs in order to record LFPs. One electrode was intra-cortically placed in the center of the RFA while a second reference electrode was placed on the overlying dura (see section Ischemic lesion and electrodes positioning), thus allowing to detect local electrical activity between the two sites. We used the same configuration in previous studies performed in our laboratory [76, 77]. We have not specifically measured spatial selectivity in the motor cortex, but from experiments on retinotopy in the visual system, we can conclude that this bipolar configuration integrates signals from neurons within a few hundred micrometers from the electrode ([76, 77] and our unpublished data), thus allowing to sample the whole RFA [15].

If PMAs play a key role in post-stroke spontaneous motor recovery, as shown in the monkey [11], electrophysiological changes in the RFA should correlate with functional restoration. One limitation of the present study is that no behavioral analysis of motor function was conducted in the recorded animals. However, behavioral consequences of the photothrombotic lesion have been longitudinally characterized in our recent work [39]. In particular, we know that CFA stroke results in long lasting impairments in forelimb motor function assessed with the Schallert Cylinder Test and the Gridwalk test. On the contrary, some degree of spontaneous recovery is detected in end point analysis in the skilled reaching test (starting from day 16) [39]. The high repeatability of the lesion obtained and the low variability in behavioral outcomes reasonably suggest the same effects in the experimental group of this work. Thus, days 16-23 of the present recordings correspond to a phase of partial functional restoration. We plan to widen this study with combined electrophysiological and behavioral experiments that allow to correlate neural activity with forelimb performance during post-stroke motor recovery.

In this study we used Granger analysis to investigate effective connectivity between the two RFAs, representing direct or indirect causal influences of one region on the other [26]. Granger causality assumes that the time series can be modeled by an autoregressive model. This is a strong assumption that can be relaxed using other methods (for example Transfer Entropy [26]), and will deserve future investigations. However we decided to use Granger analysis because it is widely accepted and often employed in the field of LFP recordings and brain connectivity (see for a review [26]).

## Supporting Information

S1 Text Intra-cortical microstimulation and optogenetic motor evoked potentials. (PDF)

S2 Text Artifacts removal algorithm. (PDF)

S3 Text Validation on artificial models of the artifact removal algorithm. (PDF)

S4 Text Definition of Granger Causality Coefficients. (PDF)

S1 FigSchematic representation of the artifact removal algorithm in a case of overlapped artifacts.The main steps, i.e identification and removal of artifacts as well as the “joining” procedure, are illustrated. A) *Z*-scored signals for both ipsi-lesional and contra-lesional hemispheres are shown. B) Peaks are identified by setting a suited threshold value: Γ = 3 and |*Z*| > Γ. C) The algorithm selects an interval *I*_*ij*_ where the artifacts are present. D) The parts of the signals inside the interval *I*_*ij*_ were removed. E) Alternative signal rearranging procedure “Minimum Method”. Points and times ($Z^{\text{ipsi}}\left(\tau_{min}^{\text{ipsi}}\right),\tau_{min}^{\text{ipsi}}$) and ($Z^{\text{contra}}\left(\tau_{min}^{\text{contra}}\right),\tau_{min}^{\text{contra}}$) of minimum distance with respect to the starting point of the artifacts $Z^{\text{ipsi}}\left(\tau_{ij}^{\text{start}}\right)$ and $Z^{\text{contra}}\left(\tau_{ij}^{\text{start}}\right)$ are shown, respectively. The green box area highlights the *N*^*r*^ points that remain after the minimization procedure. F) Graphical representation of the last part of the algorithm: the new points $Z_{\text{new}}^{\text{ipsi}}\left(\tau_{ij}^{\text{start}}+\Delta t\right)$ and $Z_{\text{new}}^{\text{contra}}\left(\tau_{ij}^{\text{start}}+\Delta t\right)$ are indicated by arrows, respectively.(TIF)

S2 Fig The removing of artifacts restores the values of the used measure. In all panels the quantities calculated for artificial models (artificial models with added artifacts) are plotted in blue (red). The results for the clean data using the “Minimum Method” (“Mean Method” with *N* = 1) are plotted in light green (green). We omitted the results for the “Mean Method” with *N* = 3,5 since produce similar results to the case *N* = 1. For all the considered measures (power bands, cross correlation, mutual information, granger causality) the effects of the presence of artifacts and the effectiveness of the algorithm to remove them are clearly visible. A) Spectral bands calculated from the autoregressive model (AR) defined in S4 Text. In the *x*-axis are reported the spectral bands *λ* of interest: *δ* = (0.5 − 4)Hz, *θ* = (4 − 8) Hz, *α* = (8 − 12) Hz, *β* = (12 − 30) Hz, *γ* = (30 − 50) Hz. On the *y*-axis the mean value and standard errors (over different noise realizations) of the relative power of each spectral band *P*_rel_(*λ*) are plotted. B) Mean and standard errors values (over different realizations) of the cross correlation by using Eq (2) (*ρ*_numeric_) for a pair of correlated gaussian random variables (of known cross correlation *ρ*_theoretical_). C) Mean and standard errors values (over different realizations) of the mutual information estimated by using the binning method Eq (4) (*I*_numeric_) for a pair of correlated gaussian random variables (of known mutual information *I*_theoretical_ as described in S2 Text). D) Values of Granger causality calculated from the signals generated by equation presented in S3 Text filtered in the frequency range (0.5 − 50) Hz against the coupling amplitude. The corresponding results are presented as mean and standard errors over different noise realizations.
(TIF)
