# Supplementary material for: Post-Stroke Longitudinal Alterations of Inter-Hemispheric Correlation and Hemispheric Dominance in Mouse Pre-Motor Cortex
Source: PLoS One. 2016 Jan 11;11(1):e0146858. doi: 10.1371/journal.pone.0146858 (PMC4709093; doi:10.1371/journal.pone.0146858)
Supplement: S1 Text — (PDF) [file pone.0146858.s001.pdf]

Supporting Information

S1 Text

Intra-cortical microstimulation and optogenetic motor evoked potentials. Mice were anesthetized with an initial cocktail of ketamine (100 mg/kg, i.p.) and xylazine (10 mg/kg, i.p.) that was supplemented with additional doses to maintain the plane of anesthesia. Each animal was then placed in a stereotaxic apparatus and a midline incision was made. The body of the animal was leaning on a support up to the chest while the forelimbs protruded from the support and were left to suspend freely. A craniotomy was performed going from 4 mm anterior to 4 mm posterior to bregma and from midline to 3 mm lateral. The dura mater was left intact and continuously moistened with saline. A tungsten microelectrode (1 MΩ, FHC, USA) was positioned in a 3 axis micromanipulator and inserted slowly in the brain at 700 μm of depth for each stimulation point. The antero-posterior and medio-lateral coordinates of each point were established following a grid with nodes spaced 250 μm. Sites of stimulation corresponded to grid intersections, although minor adjustments were sometimes necessary to avoid surface vasculature. Penetrations continued until the entire extent of forelimb motor representation was covered, including both the CFA and the RFA. At each penetration, a 40 ms train of 13 200 μs monophasic cathodal pulses was delivered at 350 Hz from an electrically isolated, constant current stimulator (World Precision Instruments Inc., USA) driven by a electronic board (BNC-2090, National Instruments Corp, USA) through a custom-made interface implemented in Lab-View (National Instruments Corp, USA) at a rate 1 Hz. The electrical circuit was closed to the same stimulator through a ground pin connected to a ground electrode placed under the skin of the neck of the animal. The amplitude of the pulses was set by the experimenter and it was increased from a minimum of 20 to a maximum of 60 μA until a visible movement was evoked in at least one body part contra-lateral or ipsi-lateral to the stimulation hemisphere. Movements were collected by a second

experimenter that was blind to the stimulation coordinate in the grid. When forelimb movement was evoked at the maximum stimulation intensity, we gradually decreased the stimulation until the movement disappeared and set the threshold current at that value, i.e. the lowest stimulation intensity required to elicit a movement. If no movement was evident at 60 μA, the site was considered non-responsive. The first penetration was performed always in the center of the CFA - where we expected to see a movement of the contra-lateral forelimb at low current threshold - in order to test both the system and the responsiveness of the animal (i.e. state of anesthesia). Once verified the responsiveness of the animal, the mapping of the entire forelimb motor cortex was performed. At the end of the mapping, the animal was sacrificed by means of an overdose of chloral hydrate.

For the optogenetic stimulation experiments, we used Thy1-ChR2 Transgenic mice (B6.Cg-Tg (Thy1-ChR2/EYFP)18Cfng/J, Jackson Laboratories, USA) that express the gene encoding for the protein ChR2 under the Thymus cell antigen-1 (Thy-1) promoter [1]. Briefly, optogenetic stimulation was delivered in anesthetized animals by means of PlexBright Optogenetic Stimulation System (Plexon Inc, USA) with a PlexBright LD-1 Single Channel LED Driver (Plexon Inc, USA) and a 456 nm Table-top LED Module connected to a 200 μm Core 0.39 NA optic fiber (Thorlabs Inc, USA). The value of the radiated light power used during the experiments is reported in terms of the percentage of the maximum power ($P_{\max} = 10$ mW) that could be emitted by the optic fiber, measured at the beginning of the experiment by an optical power meter (PM100D, Thorlabs Inc, USA). The maximum radiated power was also measured at the end of the tests with the animal, to verify that its value remained stable throughout the experimental session. Animals were deeply anesthetized with an initial cocktail of ketamine (100 mg/kg, i.p.) and xylazine (10 mg/kg, i.p.) that was supplemented with additional doses to maintain the plane of anesthesia. Mice were then shaved on the head and on both the forelimbs, up to the shoulders. A small incision was performed on both proximal forelimbs to visualize the Triceps Brachii muscle where custom-made bipolar concentric electrodes were inserted and connected to a 2-channels extracellular amplifier (EXT02F/2, Npi electronic, Germany). Electromyographic (EMG) signals were 3-500 Hz band-pass filtered and 10,000X amplified. Then, they were acquired and digitized by a OmniPlex Neural Data

Acquisition Systems (Plexon Inc, USA), with a 1 kHz sampling frequency. The animal was then placed in a stereotaxic apparatus and a midline incision was made. The body of the animal was leaning on a support up to the chest while the forelimbs protruded from the support and were left suspended freely as in ICMS experiment. A craniotomy was performed with a dental drill to expose the centre of the RFA (mm from Bregma: +2 anterior, +1.25 lateral.). The tip of the optic fiber was positioned stereotactically over the dura mater of the RFA. Optical stimulation was performed by a PlexBright single-channel LED driver (LD-1, Plexon Inc, USA) and triggered by a custom-made software developed in LabWindows CVI (National Instruments, USA) running on a PC. The LED driver was connected to the PC by means of a USB DAQ board (NI USB-6212 BNC, National Instruments, USA). Trigger events were also directed to and acquired by the OmniPlex Neural Data Acquisition Systems (Plexon Inc, USA) for the purpose of synchronizing optical stimuli with EMG responses. Muscular activity was recorded at increasing stimulation powers, i.e. starting from 5 % of maximum light power ($P_{\max}$) until the amplitude of muscular response reached a plateau, with 5% steps. Stimulation pulses were repeated 10 times for each power value, with a 5 s inter-pulse interval. At the end of the experiment, the animal was sacrificed by means of an overdose of chloral hydrate.

References

1. Hira R, Ohkubo F, Tanaka YR, Masamizu Y, Augustine GJ, Kasai H, et al. In vivo optogenetic tracing of functional corticocortical connections between motor forelimb areas. Front Neural Circuits. 2013 jan;7:55.
