# Supplementary material for: Post-Stroke Longitudinal Alterations of Inter-Hemispheric Correlation and Hemispheric Dominance in Mouse Pre-Motor Cortex
Source: PLoS One. 2016 Jan 11;11(1):e0146858. doi: 10.1371/journal.pone.0146858 (PMC4709093; doi:10.1371/journal.pone.0146858)
Supplement: S2 Text — (PDF) [file pone.0146858.s002.pdf]

Supporting Information

S2 Text

Artifacts removal algorithm.

We implemented an algorithm to clean artifacts from LFPs. The algorithm consisted in the following steps:

First, signals were normalized to have zero mean and unit standard deviation, i.e

$$Z = \frac{X - \bar{X}}{\sigma_X} \tag{1}$$

where X stands for both X^{ipsi} and X^{contra} ($\bar{X}, \sigma_X$ are the mean and the standard deviation of the signal, respectively). The Z-scored signal was then inspected to search for time intervals where the condition

$$|Z(t_i)| > \Gamma \qquad t_i = 0, \Delta t, ..., (N-1)\Delta t \tag{2}$$

holds. Γ is a parameter specifying the threshold to identify high amplitude deviations of the signal, i.e. peaks (see S1 Fig A and B).

The i-th peak was characterized by means of its starting and ending time points $\{t_i^{\text{start}}, t_i^{\text{end}}\}$, respectively (see S1 Fig C). The value of t_i^{start} is defined as the first point, crossing the threshold with positive slope, satisfying $|Z(t_i^{\text{start}})| > \Gamma$. Similarly, t_i^{end} is defined as the last point before the crossing of the threshold with negative slope.

We proceeded by identifying and then removing the i-th artifact through a procedure consisting of the following steps:

1. We identified the start and end-point of the i-th artifact τ_i^{start} and τ_i^{end} for both ipsi-lateral and contra-lateral LFP signals, through a search of the first local minima from the i-th peak

$$\tau_i^{\text{start}} := \min_{t_{i-1}^{\text{end}} \leq t \leq t_i^{\text{start}}} |Z(t)|, \tag{3}$$

$$\tau_i^{\text{end}} := \min_{t_i^{\text{end}} \leq t \leq t_{i+1}^{\text{start}}} |Z(t)|. \tag{4}$$

In this way, we identified the temporal interval $I_i = [\tau_i^{\text{start}}, \tau_i^{\text{end}}]$ of the i-th artifact.

The results of this procedure for ipsi-lesional and contra-lesional recordings are the following intervals $I_{\text{ipsi},i} = [\tau_{\text{ipsi},i}^{\text{start}}\ , \tau_{\text{ipsi},i}^{\text{end}}]$ and $I_{\text{contra},j} = [\tau_{\text{contra},j}^{\text{start}}\ , \tau_{\text{contra},j}^{\text{end}}]$, respectively.

When the intervals were overlapped, we chose a common interval $I_{ij} = [\tau_{ij}^{\text{start}}, \tau_{ij}^{\text{end}}]$ defined through the following relations :

$$\tau_{ij}^{\text{start}} := \min(\tau_{\text{ipsi},i}^{\text{start}}\ , \tau_{\text{contra},j}^{\text{start}}), \tag{5}$$

$$\tau_{ij}^{\text{end}} := \max(\tau_{\text{ipsi},i}^{\text{end}}\ , \tau_{\text{contra},j}^{\text{end}}), \tag{6}$$

(see S1 Fig C). Then, all parts of ipsi-lateral and contra-lateral signals within I_{ij} were removed (see S1 Fig D).

On the other hand, if the i-th, j-th intervals were not overlapped, we just remove the parts of signals, for both ipsi-lateral and contra-lateral recordings, inside the i-th and j-th intervals.

2. For simplicity let us consider the case of overlapped intervals. The last step, after the removal of the artifact, is to join the value of the signals at time points $\tau_{\text{i,j}}^{\text{start}}$ and $\tau_{\text{i,j}}^{\text{end}}$, both in ipsi-lateral and contra-lateral signals.

   Thus, we averaged N values of the signals before $\tau_{\text{i,j}}^{\text{start}}$ and after $\tau_{\text{i,j}}^{\text{end}}$. Then two new points $Z_{\text{ipsi}}^{\text{new}}$ and $Z_{\text{contra}}^{\text{new}}$ are added at $t = \tau_{\text{i,j}}^{\text{start}} + \Delta t$, in the ipsi-lateral and contra-lateral signals respectively. In the case of ipsi-lateral signal, the new point

is defined as follows:

$$Z_{\text{ipsi}}^{\text{new}}(\tau_{\text{ipsi},i}^{\text{start}} + \Delta t) = \sum_{n=1}^{N} \frac{Z(\tau_{\text{ipsi},i}^{\text{start}} - (n-1)\Delta t) + Z(\tau_{\text{ipsi},i}^{\text{start}} + (n-1)\Delta t)}{2N}. \quad (7)$$

The the new point $Z_{\text{contra}}^{\text{new}}$ in the contra-lateral signal is defined similarly.

We called this last ”joining” method as ”Mean Method”, which was also applied in case of non-overlapping intervals. To investigate whether the process of signals ”joining” would impact in our analysis, the Mean Method and a more sophisticated procedure, ”Minimum Method”, were compared.

The Minimum Method has been implemented based on the idea to rearrange the signal as smooth as possible. In case of overlapped artifacts, we calculated the times where the minimum difference between the signals after τ_{ij}^{end} (see eq. 5) is reached, i.e.

$$\tau_{\text{min}}^{\text{ipsi}} := \min_{\tau_{ij}^{\text{end}} \le t \le \tau_{ij}^{\text{end}} + \delta_{ij}} |Z^{\text{ipsi}}(\tau_{ij}^{\text{start}}) - Z^{\text{ipsi}}(t)| \quad , \quad (8)$$

$$\tau_{\text{min}}^{\text{contra}} := \min_{\tau_{ij}^{\text{end}} \le t \le \tau_{ij}^{\text{end}} + \delta_{ij}} |Z^{\text{contra}}(\tau_{ij}^{\text{start}}) - Z^{\text{contra}}(t)| \quad , \quad (9)$$

(see S1 Fig E) where the search of a minimum after the time τ_{ij}^{end} is over a times $\delta_{ij} = \tau_{ij}^{\text{end}} - \tau_{ij}^{\text{start}}$ equal to the duration of the artifact.

Two possible cases can occur: $\tau_{\text{min}}^{\text{ipsi}} < \tau_{\text{min}}^{\text{contra}}$ or $\tau_{\text{min}}^{\text{ipsi}} \ge \tau_{\text{min}}^{\text{contra}}$.

Let us suppose $\tau_{\text{min}}^{\text{ipsi}} < \tau_{\text{min}}^{\text{contra}}$ (the other case is analogue replacing ipsi→contra), the new value for the contra-lateral signal following the starting of the artifact is

$$Z_{\text{new}}^{\text{contra}}(\tau_{ij}^{\text{start}} + \Delta t) = Z^{\text{contra}}(\tau_{\text{min}}^{\text{contra}}). \quad (10)$$

(see S1 Fig F, bottom panel). For the ipsi-lateral signal the minimum appeared after than contra-lateral one, therefore a number of point $N^r = (\tau_{min}^{contra} - \tau_{min}^{ipsi} + \Delta t)/\Delta t$ (see green box of S1 Fig E) have also to be considered. To maintain an high level of smoothness of the signals joining, we decided to consider an average between the point of minimum distance and the mean of the remaining N^r value, i.e

$$Z^{\text{ipsi}}_{\text{new}}(\tau^{\text{start}}_{ij} + \Delta t) = \frac{Z^{\text{ipsi}}(\tau^{\text{ipsi}}_{\text{min}}) + \bar{Z}^{Nr}}{2} \tag{11}$$

(see S1 Fig F, top panel) where $\bar{Z}^{N^r}$ is defined as

$$\bar{Z}^{N^r} := \frac{\sum_{i=1}^{N^r} Z(t_i)}{N^r} \quad , \qquad t_i = \tau^{\text{ipsi}}_{\text{min}} + \Delta t, ..., \tau^{\text{contra}}_{\text{min}}. \tag{12}$$

Finally, we filtered the signals between $(0.5 - 50)$ Hz to limit the analysis to the spectral range of our interest.

When tested on artificial time series, both Mean Method and Minimum Method were equally efficient, indicating that this step is not crucial for our artifact removal algorithm (see Supporting information S3 Text and S2 Fig). However we opted for the Minimum Method to analyse our real LFP data.

S1 Fig

Schematic representation of the artifact removal algorithm in a case of overlapped artifacts. The main steps, i.e identification and removal of artifacts as well as the "joining" procedure, are illustrated. A) Z-scored signals for both ipsi-lesional and contra-lesional hemispheres are shown. B) Peaks are identified by setting a suited threshold value: $\Gamma = 3$ and $|Z| > \Gamma$. C) The algorithm selects an interval I_{ij} where the artifacts are present. D) The parts of the signals inside the interval I_{ij} were removed. E) Alternative signal rearranging procedure "Minimum Method". Points and times $(Z^{\text{ipsi}}(\tau^{\text{ipsi}}_{min}), \tau^{\text{ipsi}}_{min})$ and $(Z^{\text{contra}}(\tau^{\text{contra}}_{min}), \tau^{\text{contra}}_{min})$ of minimum distance with respect to the starting point of the artifacts $Z^{\text{ipsi}}(\tau^{\text{start}}_{ij})$ and $Z^{\text{contra}}(\tau^{\text{start}}_{ij})$ are shown, respectively. The green box area highlights the N^r points that remain after the minimization procedure. F) Graphical representation of the last part of the algorithm: the new points $Z^{\text{ipsi}}_{\text{new}}(\tau^{\text{start}}_{ij} + \Delta t)$ and $Z^{\text{contra}}_{\text{new}}(\tau^{\text{start}}_{ij} + \Delta t)$ are indicated by arrows, respectively.
