# Supplementary material for: Post-Stroke Longitudinal Alterations of Inter-Hemispheric Correlation and Hemispheric Dominance in Mouse Pre-Motor Cortex
Source: PLoS One. 2016 Jan 11;11(1):e0146858. doi: 10.1371/journal.pone.0146858 (PMC4709093; doi:10.1371/journal.pone.0146858)
Supplement: S3 Text — (PDF) [file pone.0146858.s003.pdf]

Supporting Information

S3 Text

Validation on artificial models of the artifact removal algorithm. We tested the algorithm to remove artifacts on artificial signals by using different rearranging procedures. In particular, we used both the ”Mean Method” with three different values of $N = 1, 3, 5$ and ”Minimum Method” (see S2 Text) . The signals were generated by using models for which the value of a specific measure (power band, cross correlation, mutual information, granger causality) can be computed analitically. Thus, we could estimate the effects of adding a large number of artifact recordings ($N_a = 100$) to the artificial model, as well as the efficiency of the method to remove them. To test the performance of the algorithm on spectral bands (see Spectral Analysis section in the Main Manuscript) we considered the following autoregressive model

$$x_{n+1} = a_1 x_n + \sigma \xi_n, \quad (1)$$

where ξ_n is a gaussian white noise with zero mean and unit variance. It can be shown that the variance of x is $\sigma_x^2 = \sigma^2/(1 - a_1^2)$ and its autocorrelation is proportional to $e^{-\lambda k}$ with $\lambda = -\log a_1$. In particular, the power spectrum has the form

$$P(f) = \frac{1}{\sqrt{2\pi}} \left(\frac{\sigma}{1 - a_1^2 - 2a_1 \cos(2\pi f)} \right). \quad (2)$$

For fixed initial condition $x_0 = 1$ and parameters $a_1 = 1/2$, $\sigma = 0.1$, we considered ten realizations of the autoregressive model with independent noise realizations. Each realization had a length of $N = 3 \times 10^5$ data points (the first 500 data points were neglected) and the spectrum was estimated using the Welch method of modified periodograms. The length of the windowing was set equal to $N_p = 4096$. As can be seen in the panel A of S2 Fig, the artifacts strongly affected the values of the power of spectral bands $\delta, \theta, \alpha, \beta, \gamma$ and after the application of the artifact removing algorithm

the true values are recovered (independently of the rearranging procedure).

To validate the algorithm for correlation parameters we considered the case of two gaussian variables with known correlation coefficient and mutual information. Indeed, for a fixed value of the cross correlation coefficient ρ (see Cross Correlation section in the Main Manuscript) the mutual information assumes a value expressed by the following relation

$$I = -\frac{1}{2}\log(1-\rho^2). \tag{3}$$

Then, for a fixed value of $\rho = 0.1, 0.2, ..., 0.9$, we generated ten realizations of a bivariate signal, each of length $N = 3 \times 10^4$, and both cross correlation and mutual information were estimated. The mutual information was calculated through the binning method (see Mutual Information section in the Main Manuscript) using a number of bins $N_b = 30$. In the panels B and C of S2 Fig are shown the results for the cross correlation and mutual information, respectively. The artifacts clearly altered the theoretical values of cross correlation and mutual information. Again, the results show that the algorithm to remove the artifacts is an efficient method in restoring the properties of the original signals.

Lastly, we have verified that the algorithm works well also for the case of the Granger causality measure. To this aim we considered a minimal causal autoregressive model VAR(1) described by the following set of equation:

$$\begin{aligned} x_{n+1} &= ax_n + cy_n + \xi_{x,n} \\ y_{n+1} &= by_n + \xi_{y,n} \end{aligned} \tag{4}$$

where $|a| < 1$, $|b| < 1$ and $\xi_{x,n}, \xi_{y,n}$ are unit-variance uncorrelated white noise. The parameter $c > 0$ can be viewed as a coupling constant between the two time series x, y. In additon, it can be shown that for this model the analytical expression of the Granger causality measure can be calculated $[1, 2]$

$$G(b,c)_{x\to y} = \log\left[\frac{1}{2}\left(1 + b^2 + c^2 + \sqrt{(1+b^2+c^2)^2 - 4b^2}\right)\right]. \tag{5}$$

Then, for fixed initial conditions $(x_0, y_0) = (1, 1)$ and $a = 0.3$, $b = -0.8$, ten realizations of bivariate time series were generated from the above model for each value of c ($c = 0, 0.05, ..., 0.5$) through independent noise realization. The length of each realization was $N = 3 \times 10^5$ data points (the first 500 data points were neglected) and a windowing procedure was adopted with $N_w = 512$ data points for each window. To remove the artifacts we filtered the time series within the frequency range $(0.5 - 50)$ Hz. As demonstrated by Barnett in [1], the behaviour of Granger causality under filtering changes in practical application (despite to its theoretical invariance). However, filtering is an inevitable operation in (almost every) real data experiments because of the presence of recording artifacts and non-stationarities. Thus, the values of Granger causality for a VAR(1) process filtered in the frequency range $(0.5 - 50)$ Hz was numerically computed. As stated in [1], an informed guess of model order performs better than Aikake, BIC or CV statistical test, thus following the results obtained by [1] we chose the value of $p = 16$ for each window.

In the panel D of figure S2 Fig are plotted the values of the Granger causality for the VAR(1) process filtered in the frequency range $(0.5 - 50)$ Hz and the corresponding quantity for the signal with artifacts and signal with removed artifacts.

Also in this case, the artifacts strongly affect the values of the Granger causality measure, whereas the original values of this quantity are recovered after the application of the algorithm of artifacts removal.

S2 Fig

The removing of artifacts restores the values of the used measure. In all panels the quantities calculated for artificial models (artificial models with added artifacts) are plotted in blue (red). The results for the clean data using the "Minimum Method" ("Mean Method" with $N = 1$) are plotted in light green (green). We omitted the results for the "Mean Method" with $N = 3, 5$ since produce similar results to the case $N = 1$. For all the considered measures (power bands, cross correlation, mutual information, granger causality) the effects of the presence of artifacts and the effectiveness of the algorithm to remove them are clearly visible. A) Spectral bands calculated from the autoregressive model (AR) defined by eq. 1. In the x-axis are

reported the spectral bands λ of interest: $\delta = (0.5 - 4)$Hz , $\theta = (4 - 8)$ Hz, $\alpha = (8 - 12)$ Hz, $\beta = (12 - 30)$ Hz , $\gamma = (30 - 50)$ Hz. On the y-axis the mean value and standard errors (over different noise realizations) of the relative power of each spectral band $P_{\mathrm{rel}}(\lambda)$ are plotted .

B) Mean and standard errors values (over different realizations) of the cross correlation by using eq. (1) presented in the Main Manuscript (ρ_{numeric}) for a pair of correlated gaussian random variables (of known cross correlation $\rho_{\mathrm{theoretical}}$).

C) Mean and standard errors values (over different realizations) of the mutual information estimated by using the binning method eq. (4) presented in the Main Manuscript (I_{numeric}) for a pair of correlated gaussian random variables (of known mutual information $I_{\mathrm{theoretical}}$ as described by eq. 3).

D) Values of Granger causality calculated from the signals generated by eq.4 filtered in the frequency range $(0.5 - 50)$ Hz against the coupling amplitude. The corresponding results are presented as mean and standard errors over different noise realizations.

References

1. Barnett L, Seth AK. Behaviour of Granger causality under filtering: Theoretical invariance and practical application. J Neurosci Methods. 2011;201(2):404–419.

2. Barnett L, Seth AK. The MVGC multivariate Granger causality toolbox: a new approach to Granger-causal inference. J Neurosci Methods. 2014 feb;223:50–68.
