# Supplementary material for: Post-Stroke Longitudinal Alterations of Inter-Hemispheric Correlation and Hemispheric Dominance in Mouse Pre-Motor Cortex
Source: PLoS One. 2016 Jan 11;11(1):e0146858. doi: 10.1371/journal.pone.0146858 (PMC4709093; doi:10.1371/journal.pone.0146858)
Supplement: S4 Text — (PDF) [file pone.0146858.s004.pdf]

Supporting Information

S4 Text

Definition of Granger Causality Coefficients.

The two signals $x(t_i)$, i.e. ipsi-lateral LFP) and $y(t_i)$, i.e.contra-lateral LFP, (sampled at $t_i = 0, \Delta t, ..., (N-1)\Delta t$, where $N = 3.6 \times 10^5$) are modelled by a univariate autoregressive models of order p

$$x(t_i) \quad := \quad \sum_{k=1}^{p} a_k x(t_{i-k}) + \nu_1(t_i), \tag{1}$$

$$y(t_i) \quad := \quad \sum_{k=1}^{p} b_k y(t_{i-k}) + w_1(t_i), \tag{2}$$

or by a bivariate autoregressive model

$$x(t_i) \quad := \quad \sum_{k=1}^{p} c_k x(t_{i-k}) + \sum_{k=1}^{p} d_k y(t_{i-k}) + \nu_2(t_i), \tag{3}$$

$$y(t_i) \quad := \quad \sum_{k=1}^{p} e_k y(t_{i-k}) + \sum_{k=1}^{p} f_k x(t_{i-k}) + w_2(t_i), \tag{4}$$

where $a_k, b_k, c_k, d_k, e_k, f_k$ are the coefficients of the autoregressive models and ν_1, w_1, ν_2, w_2 the prediction errors or residual. The coefficients of the models and the sum of the square residuals were estimated by solving Yule-Walker equations [1].

In the following, we are interested to statistically test whether the time series $y(t_i)$ helps the predicting of the $x(t_i)$ signal or not, i.e. whether the following null hypothesis H_0

$$H_0 : d_1 = d_2 = ... = d_p = 0. \quad (5)$$

can be rejected or not. Then, It can be shown that the variable

$$G_{y \to x} = \frac{(N-2p)}{p}\big[\frac{R_{x,0}}{R_{x,1}} - 1\big] \quad (6)$$

is a measure of the prediction ability of $x(t_i)$ with the aid of $y(t_i)$ [1], where $R_{x,0}(t_i) = \sum_{i=0}^{N-1} \nu_1^2(t_i)$ and $R_{x,1}(t_i) = \sum_{i=0}^{N-1} \nu_2^2(t_i)$.

Moreover, it can be shown that $G_{y \to x}$ follows an $F(\gamma_1, \gamma_2)$ distribution with degree of freedoms $\gamma_1 = p$ and $\gamma_2 = N - p$ and this can be used to test whether H_0 can be rejected with an assigned probability level α (we chose $\alpha = 0.05$). Similarly, the values of

$$G_{x \to y} = \frac{(N-2p)}{p}\big[\frac{R_{y,0}}{R_{y,1}} - 1\big] \quad (7)$$

can be used to assess whether $x(t_i)$ helps the prediction of $y(t_i)$, where $R_{y,0}(t_i) = \sum_{i=0}^{N-1} w_1^2(t_i)$ and $R_{y,1}(t_i) = \sum_{i=0}^{N-1} w_2^2(t_i)$.

To avoid problems in the estimation of the autoregressive models parameters, arising from nonstationarity, a windowing of data was adopted. For each window ($N_p = 512$ data points) we estimated the order p of the autoregressive model using the Aikake and BIC criterion combined with the requirement of stability for the autoregressive model [2]. For our LFPs the p turned out to be 15.

A value of $p = 15$ allows us to consider an inter-hemispheric dynamics of $\Delta t * p = 75$ ms (where $\Delta t = 5$ ms is the sampling rate) which may be a reasonable value for studying the inter-hemispheric cross-talk, because, in our signals, the time scale of the inter-hemispheric dynamics ranges over an interval of about $0 - 100$ ms. We assessed this interval ($0 - 100$ ms) by observing that both cross-correlation and mutual information measures showed negligible values after 100 ms indicating that the hemispheres are uncorrelated (data not shown).

Since we are dealing with real signal, the values of granger causality $G_{y \to x}$ (or $G_{y \to x}$) could be biased by several factor. For this reason, the information of the coupling direction was established by statistical inference [3]. Thus, we quantified the

strength of the influence from $y \to x$ and from $x \to y$ by defining the probability, over windows, that the null hypothesis H_0 can be rejected, i.e

$$\begin{aligned} G^p_{y \to x} &:= \frac{N_{yx}}{N_w} \\ G^p_{x \to y} &:= \frac{N_{xy}}{N_w} \end{aligned} \tag{8}$$

where $N_{yx}(N_{xy})$ is the number of windows such that the statistic $G_{x\to y}$ $(G_{y\to x})$ was significant $G_{x\to y} > F_\alpha$ $(G_{y\to x} > F_\alpha)$ and N_w the total number of windows.

Moreover, we constructed the frequency distributions of hemispheric dominance, f_{HD}, counting the number of time windows in which ipsi $\to$ contra *dominance* $(G_{x\to y} > F_\alpha$ and $G_{y\to x} < F_\alpha)$, contra $\to$ ipsi *dominance* $(G_{y\to x} > F_\alpha$ and $G_{x\to y} < F_\alpha)$ or *balance* $(G_{y\to x} > F_\alpha$ and $G_{x\to y} > F_\alpha$, otherwise, $G_{y\to x} < F_\alpha$ and $G_{x\to y} < F_\alpha)$ occurred, and then normalized by the total number of windows.

References

1. Gourévitch B, Bouquin-Jeannès RL, Faucon G. Linear and nonlinear causality between signals: methods, examples and neurophysiological applications. Biol Cybern. 2006 oct;95(4):349–69. Available from: `http://link.springer.com/10.1007/s00422-006-0098-0`.

2. Barnett L, Seth AK. The MVGC multivariate Granger causality toolbox: a new approach to Granger-causal inference. J Neurosci Methods. 2014 feb;223:50–68.

3. Antonucci F, Di Garbo A, Novelli E, Manno I, Sartucci F, Bozzi Y, et al. Botulinum neurotoxin E (BoNT/E) reduces CA1 neuron loss and granule cell dispersion, with no effects on chronic seizures, in a mouse model of temporal lobe epilepsy. Exp Neurol. 2008;210(2):388–401.
