# Supplementary figures and images for: Post-Stroke Longitudinal Alterations of Inter-Hemispheric Correlation and Hemispheric Dominance in Mouse Pre-Motor Cortex
Source: PLoS One. 2016 Jan 11;11(1):e0146858. doi: 10.1371/journal.pone.0146858 (PMC4709093; doi:10.1371/journal.pone.0146858)

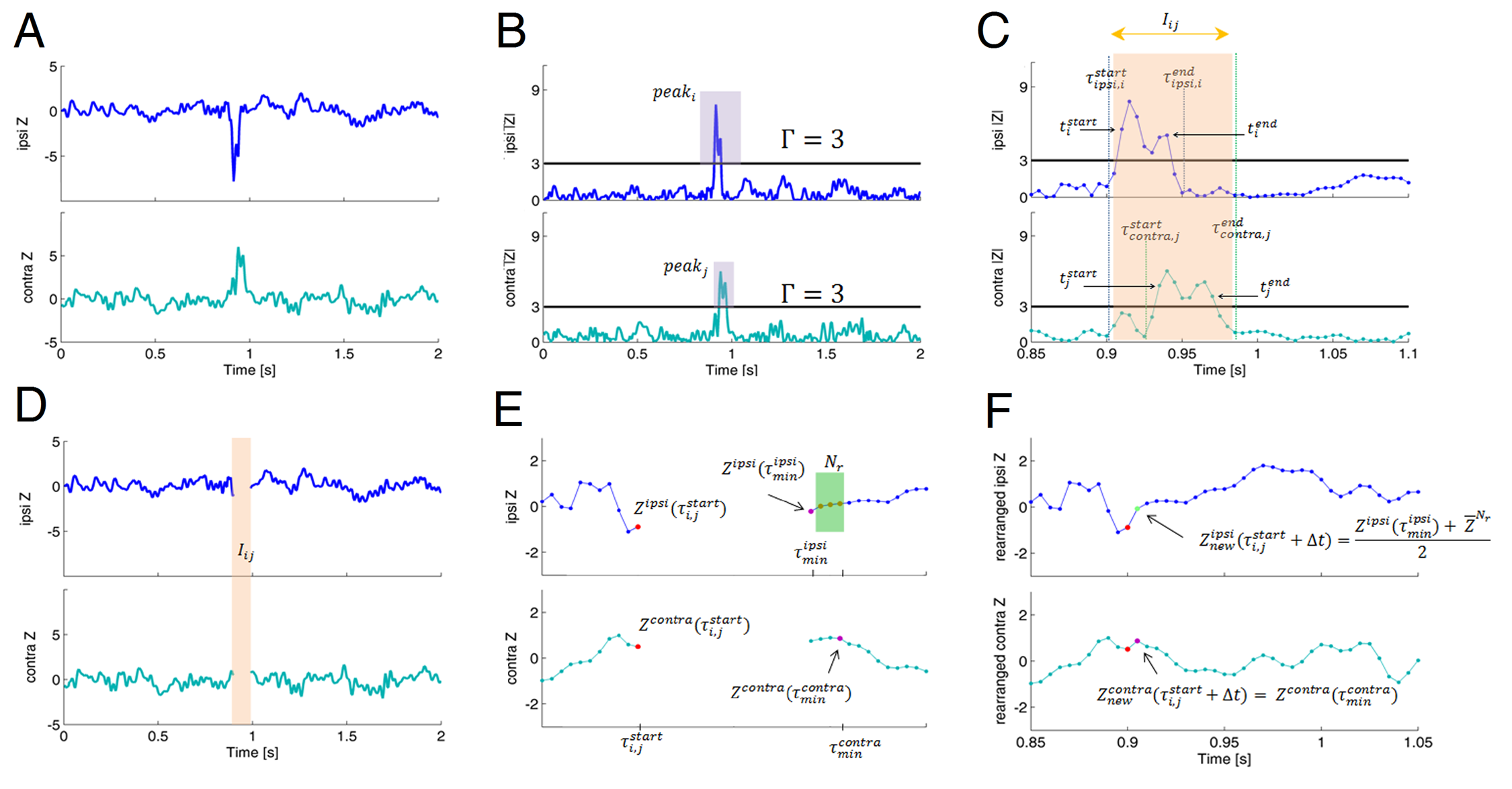

Supplement: S1 Fig — The main steps, i.e identification and removal of artifacts as well as the “joining” procedure, are illustrated. A) Z-scored signals for both ipsi-lesional and contra-lesional hemispheres are shown. B) Peaks are identified by setting a suited threshold value: Γ = 3 and |Z| > Γ. C) The algorithm selects an interval Iij where the artifacts are present. D) The parts of the signals inside the interval Iij were removed. E) Alternative signal rearranging procedure “Minimum Method”. Points and times (Zipsi(τminipsi),τminipsi) and (Zcontra(τmincontra),τmincontra) of minimum distance with respect to the starting point of the artifacts Zipsi(τijstart) and Zcontra(τijstart) are shown, respectively. The green box area highlights the Nr points that remain after the minimization procedure. F) Graphical representation of the last part of the algorithm: the new points Znewipsi(τijstart+Δt) and Znewcontra(τijstart+Δt) are indicated by arrows, respectively. (TIF) [file pone.0146858.s005.tif]

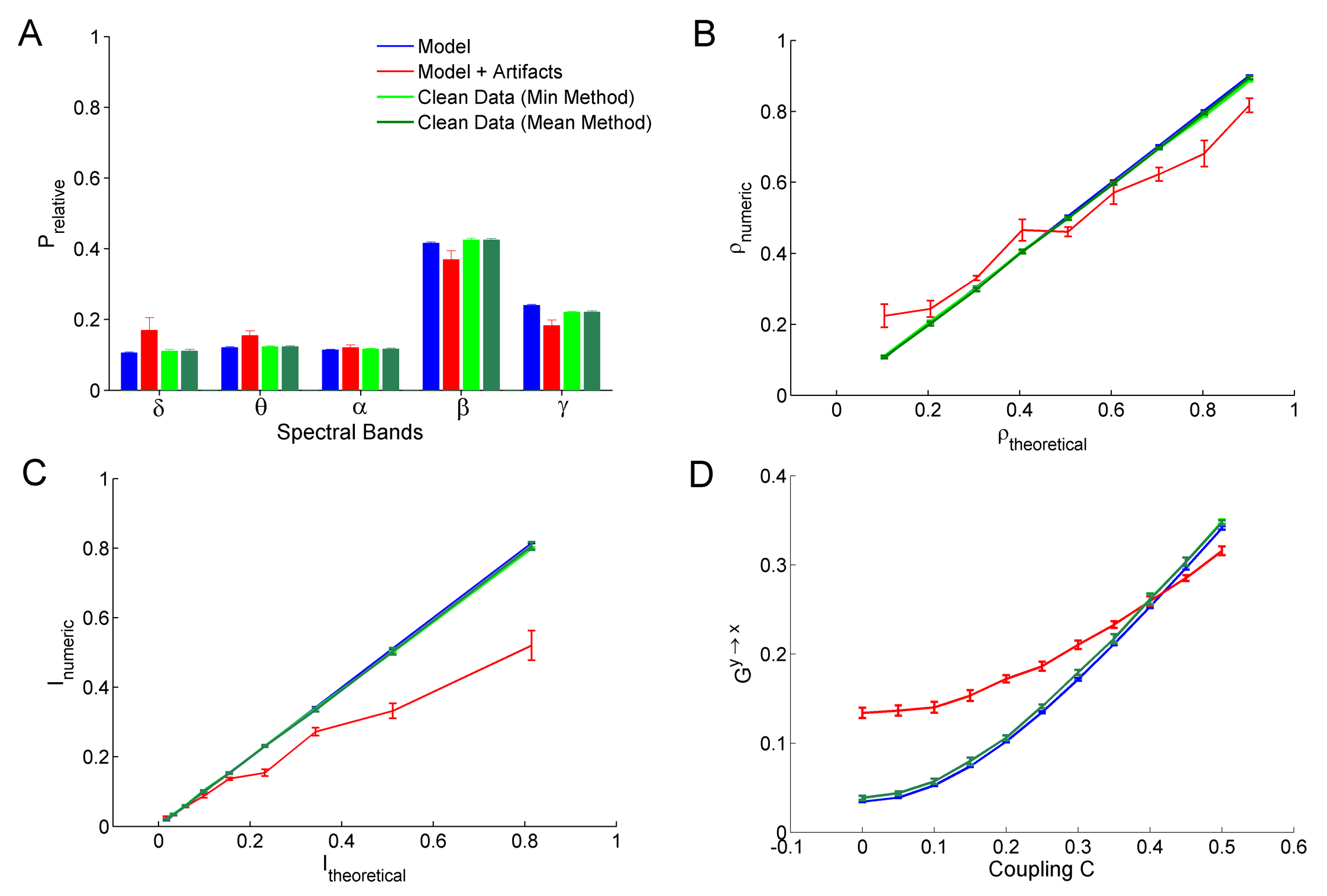

Supplement: S2 Fig — In all panels the quantities calculated for artificial models (artificial models with added artifacts) are plotted in blue (red). The results for the clean data using the “Minimum Method” (“Mean Method” with N = 1) are plotted in light green (green). We omitted the results for the “Mean Method” with N = 3,5 since produce similar results to the case N = 1. For all the considered measures (power bands, cross correlation, mutual information, granger causality) the effects of the presence of artifacts and the effectiveness of the algorithm to remove them are clearly visible. A) Spectral bands calculated from the autoregressive model (AR) defined in S4 Text. In the x-axis are reported the spectral bands λ of interest: δ = (0.5 − 4)Hz, θ = (4 − 8) Hz, α = (8 − 12) Hz, β = (12 − 30) Hz, γ = (30 − 50) Hz. On the y-axis the mean value and standard errors (over different noise realizations) of the relative power of each spectral band Prel(λ) are plotted. B) Mean and standard errors values (over different realizations) of the cross correlation by using Eq (2) (ρnumeric) for a pair of correlated gaussian random variables (of known cross correlation ρtheoretical). C) Mean and standard errors values (over different realizations) of the mutual information estimated by using the binning method Eq (4) (Inumeric) for a pair of correlated gaussian random variables (of known mutual information Itheoretical as described in S2 Text). D) Values of Granger causality calculated from the signals generated by equation presented in S3 Text filtered in the frequency range (0.5 − 50) Hz against the coupling amplitude. The corresponding results are presented as mean and standard errors over different noise realizations. (TIF) [file pone.0146858.s006.tif]
